# Supramolecular Prodrug Nanovectors for Active Tumor Targeting and Combination Immunotherapy of Colorectal Cancer

**DOI:** 10.1002/advs.201903332

**Published:** 2020-02-25

**Authors:** Xianli Hu, Bo Hou, Zhiai Xu, Madiha Saeed, Fang Sun, Zhenmei Gao, Yi Lai, Tong Zhu, Fan Zhang, Wen Zhang, Haijun Yu

**Affiliations:** ^1^ School of Chemistry and Molecular Engineering East China Normal University Shanghai 200241 China; ^2^ State Key Laboratory of Drug Research & Center of Pharmaceutics Shanghai Institute of Materia Medica Chinese Academy of Sciences Shanghai 201203 China; ^3^ Department of Chemistry Fudan University Shanghai 200438 China

**Keywords:** active tumor targeting, cancer management, colorectal cancer, combination immunotherapy, photodynamic immunotherapy, prodrugs, supramolecular nanovectors

## Abstract

Immunotherapy aiming to harness the exquisite power of the immune system has emerged as a crucial part of clinical cancer management. However, only a subset of cancer patients responds to current immunotherapy because of low immunogenicity of the tumor cells and immunosuppressive tumor microenvironment. Herein, host–guest prodrug nanovectors are reported for active tumor targeting and combating immune tolerance in tumors. The prodrug nanovectors are designed by integrating hyaluronic acid (HA) and reduction‐labile heterodimer of Pheophorbide A (PPa) and NLG919 into the supramolecular nanocomplexes, where PPa and NLG919 act as a photosensitizer and potent inhibitor of indoleamine 2,3‐dioxygenase 1 (IDO‐1), respectively. Meanwhile, HA is employed to achieve active tumor targeting by recognizing CD44 overexpressed on the surface of tumor cell membranes. Near infrared (NIR) laser irradiation triggers the release of reactive oxygen species to provoke antitumor immunogenicity and intratumoral infiltration of cytotoxic T lymphocytes (CTLs). Meanwhile, the immunosuppressive tumor microenvironment (ITM) is reversed by NLG919‐mediated IDO‐1 inhibition. Combination of photodynamic immunotherapy and IDO‐1 blockade efficiently eradicates CT26 colorectal tumors in the immunocompetent mice. The host–guest nanoplatform capable of eliciting effective antitumor immunity by inactivating inhibitory immune response can be applied to other immune modulators for improved cancer immunotherapy.

## Graphical abstract

Host–guest prodrug nanovectors integrating hyaluronic acid, and heterodimer of NLG919 and Pheophorbide A are reported for photodynamic immunotherapy of cancer. The combination of prodrug nanovector with near infrared laser irradiation triggers the generation of reactive oxygen species for eliciting antitumor immunogenicity and intratumoral infiltration of cytotoxic T lymphocytes (CTLs). Meanwhile, NLG919 activates the CTLs by relieving the immunosuppressive tumor microenvironment.

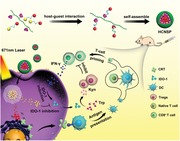

## 1 Introduction

Immune checkpoint blockade (ICB) therapy utilizing mono‐ or dual‐immune checkpoint inhibitors has emerged as one of the mainstream approaches for clinical cancer management.^[^ 1, 2, 3 ^]^ ICB therapy has shown unprecedented clinical responses to improve the survival rate of cancer patients. However, only a subset of cancer patients responds to current ICB therapy because of immune tolerance, which is most likely induced by low tumor immunogenicity and insufficient intratumoral infiltration of cytotoxic T lymphocytes (CTLs).^[^ 4, 5, 6, 7, 8 ^]^ The immune tolerance and ultimate immunosuppressive tumor microenvironment (ITM) are associated with the overactivation of the immune checkpoints (e.g., programmed cell death ligand 1 (PD‐L1) and CD47) and other negative immune regulators (e.g., indoleamine 2,3‐dioxygenase 1 (IDO‐1)) in the tumor cells.^[^ 9, 10, 11, 12 ^]^ Despite their specificity and binding affinity, the monoclonal antibody‐based immune checkpoint inhibitors may have several intrinsic disadvantages, including limited tumor penetration, immune‐related adverse effects, and inadequate pharmacokinetics.^[^ 13, 14, 15, 16, 17, 18, 19 ^]^ To improve the response rate and overcome the disadvantages of monoclonal antibody‐based immune inhibitor remain a formidable challenge in ICB therapy.

In recent years, small molecular immune modulators have attracted extensive attention for improved cancer immunotherapy. The small molecular immune modulators can be rationally designed to target multiple targets either on the tumor surfaces or in the tumor microenvironment.^[^ 20, 21, 22, 23 ^]^ Moreover, small molecules show much better tumor penetration capability than their monoclonal antibody counterparts.^[^ 4 ^]^ However, the combination of immune therapy with the small molecular immune modulators suffers from varied pharmacokinetics profiles. The small molecular compounds generally lack tumor specificity, which could induce severe side effects because of nonspecific drug distribution in the normal tissues.^[^ 24, 25, 26 ^]^ Therefore, it is highly desirable to spatiotemporally codeliver multiple immune modulators to the tumor site for combination immunotherapy. Among the various nanovectors exploited for codelivery of multiple therapeutic regimens, supramolecular nanocomplexes have shown considerable potential for targeted drug delivery and cancer therapy.^[^ 27, 28, 29, 30 ^]^ The supramolecular nanoassemblies fabricated through noncovalent host–guest interactions are capable of responding to the endogenous or exogenous stimulus for tumor‐specific drug delivery and therefore ensure tunable drug release at the intended target sites.^[^ 31, 32, 33, 34, 35, 36, 37, 38 ^]^

**Figure 1:**
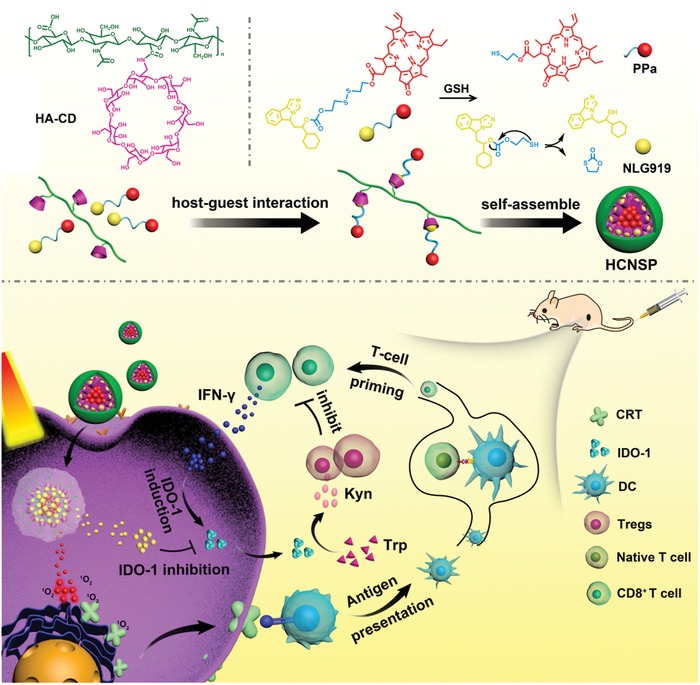
a) Fabrication of supramolecular HCNSP nanovector though host–guest complexation between HA‐CD and NSP. b) Schematic illustration of HCNSP to perform combination immunotherapy by simultaneous ICD induction and IDO‐1 inhibition.

To achieve highly efficient cancer immunotherapy, we herein designed the supramolecular prodrug nanovectors for tumor‐specific codelivery of multiple immune modulators. The nanovectors were engineered by complexing β‐cyclodextrin‐grafted hyaluronic acid (HA‐CD) with a disulfide bond‐crosslinked heterodimer of NLG919 and pyropheophorbide A (PPa) (termed as NSP) through host–guest interaction (**Figure** **1**a). PPa is a widely used photosensitizer (PS) for photodynamic therapy (PDT).^[^ 39, 40 ^]^ Meanwhile, NLG919 is a potent inhibitor of IDO‐1 for immunotherapy.^[^ 41, 42, 43 ^]^ The supramolecular nanovectors actively accumulated at the tumor site via HA‐mediated recognition of CD44 overexpressed on the surface of tumor cells.^[^ 44, 45 ^]^ Upon intracellular uptake, the prodrug nanovectors were activated inside the tumor cells via glutathione‐triggered cleavage of the disulfide bond of NSP or hyaluronidase‐mediated degradation of the HA backbone. Owing to the combination of the prodrug nanovectors with 671 nm laser irradiation, PPa induced reactive oxygen species (ROS) generation to trigger immunogenic cell death (ICD) and antitumor immune response in the tumor. Meanwhile, NLG919 suppressed the activity of IDO‐1 for combating the immune tolerance of cancer cells (Figure 1b). To our knowledge, this is the first demonstration of host–guest nanovector for active tumor targeting and combination immunotherapy.

## 2 Result and Discussion

### 2.1 Preparation and Characterization

HA‐CD was synthesized by grafting NH_2_‐β‐CD onto the backbone of HA via an amide bond between the carboxyl group of HA and the amine group of NH_2_‐β‐CD (Figure S1, Supporting Information). The successful synthesis of the HA‐CD was verified by Fourier transform infrared (FTIR) spectroscopic and ^1^H NMR spectrum examination, respectively (Figures S2 and S3, Supporting Information). The CD‐grafting ratio was determined to 43% with around half of the carboxyl groups grafted with CD as determined by integrating the characteristic proton of CD and HA, respectively. Meanwhile, the reduction‐activatable heterodimer of NLG919‐S‐S‐PPa (NSP) was synthesized by coupling NLG919 with PPa via a disulfide bond. A reduction‐insensitive analog of NSP was synthesized by conjugating NLG919 with PPa via an alkyl spacer, which was termed as NLG919‐C‐C‐PPa (NCP) (Figures S4 and S5, Supporting Information).

The chemical structure of NSP and NCP was examined by ^1^H NMR spectra and mass spectrum (MS) measurements, respectively (Figures S6–S9, Supporting Information). The supramolecular prodrug nanovectors were then prepared by complexing HA‐CD with NSP or NCP, and the resultant nanoassemblies were termed as HCNSP and HCNCP, respectively. The NLG919 and PPa concentrations in the HCNSP and HCNCP prodrug nanovectors were determined to be ≈6.1 (wt)% and ≈11.6 (wt)%, respectively.

**Figure 2:**
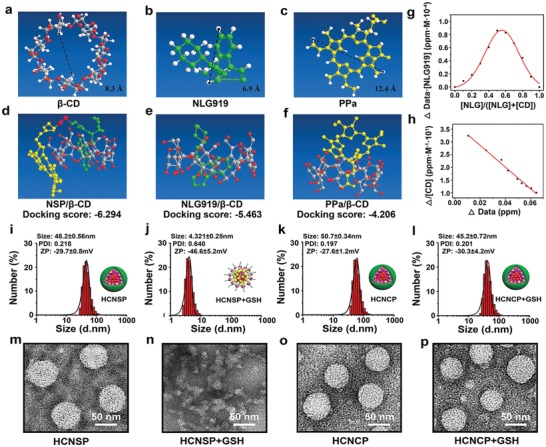
Fabrication and characterization of the supramolecular nanovectors for combination immunotherapy. a) Computer simulation of the inner cavity diameter of β‐CD. Computer simulation of the maximum diameter of b) NLG919 and c) PPa. d–f) Molecular docking simulation data between β‐CD and d) NSP, e) NLG919, or f) PPa, respectively. g) The inclusion ratio between β‐CD and NLG919 determined by Job plot method and ^1^H NMR spectra. h) The stability constant of β‐CD and NLG919 host–guest complexes in aqueous solution determined by Benese‐Hildebrand equation and ^1^H NMR spectra. DLS‐determined particle size distribution and the representative TEM image of i,m) HCNSP; j,n) HCNSP + 10 × 10^−3^ m of GSH; k,o) HCNCP; and l,p) HCNCP + 10 × 10^−3^ m of GSH (scale bar = 50 nm).

To clarify the host–guest interaction between NSP and HA‐CD, the tendency of NLG919/β‐CD or PPa/β‐CD complexation was detected by Chem‐3D simulation. The calculated maximum diameter of NLG919, i.e., 6.9 Å, was smaller than the inner cavity diameter of β‐CD (8.3 Å), whereas the maximum diameter of PPa (i.e., 12.4 Å) was much larger than the inner cavity diameter of β‐CD, which hindered the formation of the stable complex between PPa and CD (**Figure** **2**a–c). Furthermore, the molecular docking simulation was employed to determine the interaction between β‐CD and the guest molecules (e.g., NLG919, PPa, and NSP). The NSP/β‐CD host–guest complex displayed a docking score of −6.294, which was 1.15 and 1.50‐fold lower than those of NLG919/β‐CD (‐5.463) and PPa/β‐CD (‐4.206) complexes respectively, suggesting β‐CD intends to form a stable complex with NSP through host‐guest interaction with NLG919 instead of PPa (Figure 2d–f).

We further calculated the inclusion ratio between β‐CD and NLG919. The inclusion ratio (1:1) and binding constant (4.2 × 10^2^ M^−1^) between NLG919/β‐CD complexes as calculated by Job plot method and Benese‐Hildebrand equation, respectively using ^1^H‐NMR spectra, implying stable inclusion of NLG919 with β‐CD (Figure 2g,h). Therefore, the above data consistently suggested the stable complexation between β‐CD and NLG919.

Transmission electron microscopy (TEM) and dynamic light scattering (DLS) analysis demonstrated spherical morphology of HCNSP with an average hydrodynamic diameter of 48.2 ± 0.6 nm and negative surface charge of −29.7 ± 0.8 mV, respectively (Figure 2i,m). The decomposition of HCNSP with the addition of 10 × 10^−3^ m GSH, indicating the cleavage of the disulfide bonds of NSP dimer and thus verifying the superior reduction‐sensitivity of HCNSP (Figure 2j,n). In contrast, HCNCP displayed constant morphology and hydrodynamic diameter irrespective of the presence of GSH (Figure 2k,l,o,p). The nanocomplex between β‐CD and NSP (CNSP) exhibited a comparatively larger hydrodynamic diameter (1859 ± 104 nm) while lower colloidal stability than HCNSP and HCNCP as revealed by DLS, UV–vis, and fluorescence spectra, verifying the crucial role of HA for the formation of supramolecular nanoparticles and stabilization of the CNSP complex stabilize the host‐guest nanoassemblies (Figures S10–S14, Supporting Information).

**Figure 3:**
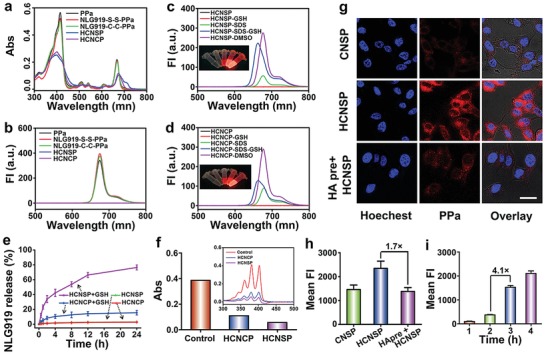
Chemophysical characterization of the prodrug nanovectors. a) UV–vis spectra of PPa, NSP, NCP, HCNSP, and HCNCP at the identical PPa concentration. b) Fluorescence spectra properties of PPa, NSP, NCP, HCNSP, and HCNCP at the identical PPa concentration. The influence of GSH and SDS incubation on the fluorescence features of c) HCNSP, and d) HCNCP, respectively (the inset showed the fluorescence imaging of two prodrug nanovectors). e) NLG919 release profile of HCNSP and HCNCP with or without incubation with 10 × 10^−3^ m GSH. f) Laser‐induced ROS generation of the HCNSP and HCNCP as determined by using ABDA as an indicator, the inset showed the UV–vis spectra characterization. g) CLSM images of intracellular distribution of CNSP, HCNSP, and HCNSP (scale bar = 25 µm). h) Flow cytometric detection of cellular uptake of CNSP, HCNSP, the cells of the HCNSP group were pretreated with free HA. i) Flow cytometric study of intracellular uptake of HCNSP in CT26 cells as a function of incubation time.

UV–vis spectroscopic study of the nanovector suspension displayed red shift of PPa absorption peak at 650 nm, implying the formation of J‐aggregates of PPa molecules in the hydrophobic core of HCNSP nanoparticles (**Figure** **3**a). The fluorescence spectrum showed that the fluorescence of HCNSP or HCNCP was completely quenched due to π–π stacking and Förster resonance energy transfer (FRET) effect between PPa molecules (Figure 3b). With the addition of 10 × 10^−3^ m GSH for disulfide bond cleavage, the fluorescence emission of PPa did not recover, which due to PPa aggregate and thus the fluorescence was quenched. The addition of sodium dodecyl sulfate (SDS) could enhance the dispersion of PPa and dramatically recovery the fluorescence emission of HCNSP (Figure 3c). In contrast, the fluorescence emission of HCNCP group moderately increased upon incubation with SDS and GSH since NLG919‐C‐C‐PPa is non‐sensitive to GSH (Figure 3d).

We next investigated GSH‐triggered NLG919 release from HCNSP and HCNCP using high performance liquid chromatography (HPLC). About 80% of NLG919 was released from HCNSP when incubated with 10 × 10^−3^ m GSH while less than 15% NLG919 release from HCNCP implying that GSH significantly promote the decomposition of HCNSP and thus efficiently release NLG919 (Figure 3e).

### 2.2 Photoactivity and Cellular Uptake of the Prodrug Nanovector In Vitro

The photoactivity of HCNSP was assessed by measuring ROS generation using 9,10‐anthracenediyl‐bis(methylene)‐dimalonic acid (ABDA) as the ROS indicator. Upon 671 nm laser irradiation, photodensity, concentration as well as time‐dependent decline in UV–Vis absorption of ABDA indicating irradiation‐triggered ROS generation (Figure S15, Supporting Information). HCNSP showed higher photoactivity than HCNCP, which could be most likely explained by ROS‐mediated cleavage of disulfide bonds and dissociation of the prodrug nanovectors (Figure 3f).

Next, we examined the active tumor targeting profile of HCNSP in CT26 colorectal tumor cells in vitro. CT26 tumor cells were identified for CD44 overexpressed on the surface of cell membrane.^[^ 46 ^]^ HCNSP displayed increased cellular uptake in comparison to CNSP (without HA moiety), and the cellular uptake of HCNSP was blocked when the tumor cells were pretreated with HA, suggesting the crucial role of HA‐CD44 interaction for cellular uptake of HCNSP nanoparticles. Flow cytometric analysis further revealed 1.7‐fold higher intracellular uptake of HCNSP than other groups (Figure 3g,h).

The intracellular fluorescence of the HCNSP group increased gradually in a time‐dependent manner, implying endocytosis‐dependent cellular uptake of the prodrug nanovectors (Figure 3i, Figure S16, Supporting Information).

**Figure 4:**
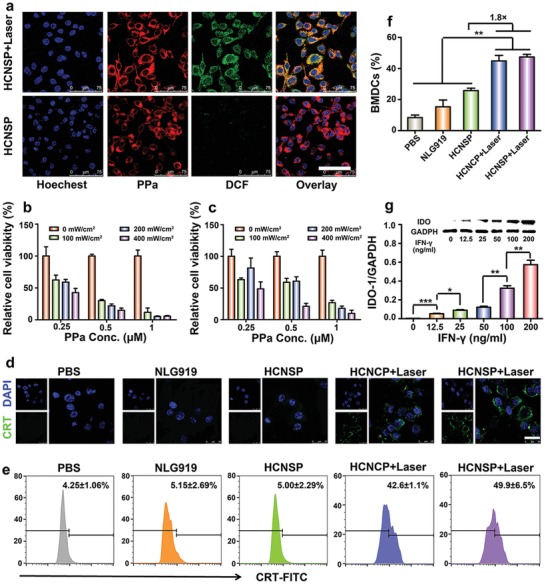
a) CLSM images of laser‐induced ROS generation of HCNSP in CT26 cells in vitro (scale bar = 75 µm). The cells were then illuminated with 671 nm laser for 5 min at photodensity of 100, 200, or 400 mW cm^−2^. The intracellular ROS generation was then determined by CLSM and flow cytometric examination of DCF fluorescence intensity. Phototoxicity assay of b) HCNSP and c) HCNCP in combination with 671 nm laser irradiation at photodensity of 100 mW cm^−2^ for 30 s, the cell viability was then examined by MTT assay after 24 h incubation. d) CLSM images of laser‐induced ICD with CRT‐FITC antibody for the analysis of CRT exposure in CT26 cell surface (scale bar = 25 µm). e) Flow cytometric detection of laser‐induced CRT exposure. f) Frequency of laser‐induced matured BMDCs by flow cytometric analysis (**p < 0.01). g) Western‐blot assay of IFN‐γ‐induced IDO‐1 upregulation in CT26 tumor cells in vitro (*p < 0.05, **p < 0.01, ***p < 0.001).

The cytotoxicity of HCNSP and HCNCP was then evaluated in CT26 cells by 3‐(4,5‐dimethylthiazol‐2‐yl)‐2,5‐diphenyltetrazolium bromide (MTT) assay. HCNSP and HCNCP groups both displayed high cell viability (i.e., more than 80%) even at a PPa concentration up to 8 × 10^−6^ m after 24 h incubation, indicating negligible cytotoxicity of the nanovectors (Figure S17, Supporting Information). NIR laser irradiation‐induced intracellular ROS generation was examined by 2′,7′‐dichlorofluorescein diacetate (DCFH‐DA). The DCFH‐DA without fluorescence could be oxidized by ROS to DCF with green fluorescence. According to the CLSM and flow cytometric analysis, verifying the strong photoactivity of nanovectors (**Figure** **4**a, Figure S18, Supporting Information).

We then tested the phototoxicity of the prodrug nanovectors in CT26 cells in vitro. The cells were incubated with HCNSP or HCNCP for 24 h and illuminated with 671 nm laser at photodensity of 100 mW cm^−2^ for 30 s, the cell viability was measured after the additional 24 h incubation. Upon laser irradiation, the cell viability of both HCNSP or HCNCP group dramatically decreased as a function of photodensity. Furthermore, laser‐triggered phototoxicity of HCNSP was twofold higher than that of HCNCP (Figure 4b,c).

### 2.3 ICD Induction and DC Maturation In Vitro

We next sought to investigate the potential of the prodrug nanovectors to induce immunogenic cell death (ICD) in CT26 tumor cells by determining membrane exposure of calreticulin (CRT) and extracellular release of high mobile group box 1 (HMGB1). CT26 cells were incubated with NLG919, HCNSP, and HCNCP for 12 h and then incubated for another 4 h after being irradiated with 671 nm laser at 100 mW cm^−2^ for 30 s. The cells were stained with Alexa 488‐anti‐CRT antibody for immunofluorescence assay. The CLSM results displayed the presence of secreted CRT on the cell membrane of HCNSP and HCNCP irradiated groups. Flow cytometric data further revealed remarkable increase of the CRT‐positive rate from 4.25% ± 1.1% to 49.9% ± 6.5%, which was almost 12‐times higher than that of the PBS group (Figure 4d,e). HMGB1 localized in the cellular nucleus of the PBS, free NLG919 and HCNSP groups. In contrast, 671 nm laser irradiation dramatically promoted >90% extracellular HMGB1 release in the HCNCP+Laser and HCNSP+Laser groups, further confirming the occurrence of ICD in the laser‐treated tumor cells (Figure S19, Supporting Information).

Dendritic cells (DCs) play a crucial role in initiating and regulating the innate and adaptive immune response. To evaluate PDT‐elicited immune response of the tumor cells, we further investigated ICD‐induced maturation of DCs in vitro. Bone marrow derived dendritic cells (BMDCs) were freshly separated from Balb/c mice and coincubated with pretreated CT26 tumor cells, and the maturation of DCs (CD11c^+^CD80^+^CD86^+^) was detected by flow cytometry. Compared with PBS, NLG919 and HCNSP could not induce obvious DCs maturation after 24 h of coincubation. However, HCNCP and HCNSP significantly induced the DCs maturation upon laser irradiation, which was about 1.8‐fold higher than that of the HCNSP group (Figure 4f).

Matured DCs can elicit antitumor immunity by presenting tumor‐specific antigens to CTLs, which induce tumor cell apoptosis by secreting proinflammatory cytokines, including interferon‐γ (IFN‐γ). Western‐blot assay confirmed that IDO‐1 expression was upregulated by IFN‐γ in CT26 tumor cells in a concentration‐dependent manner (Figure 4g). IDO‐1 can in turn ablate the therapeutic performance of photodynamic immunotherapy by inhibiting the proliferation of CTLs.^[^ 47 ^]^ It was, therefore, logical to combine photodynamic immunotherapy with IDO‐1 blockade.

IDO‐1 is highly expressed in the tumor microenvironment (TME) and responsible for catabolizing an essential amino acid, i.e., tryptophan (Trp) to kynurenine (Kyn).^[^ 48 ^]^ Kyn inhibits CTLs function by inducing T cells exhaustion and apoptosis and therefore form the ITM.^[^ 49 ^]^ To evaluate the bioactivity of NLG919‐PPa conjugate, we compared the IDO‐1 inhibition activity of HCNCP and HCNSP nanoparticles by examining endogenous Trp and Kyn concentrations in CT26 tumor cells in vitro. The results showed that HCNCP moderately inhibited ≈40% Trp activity IDO‐1, which could be most likely explained by slow release of NLG919 from HCNCP nanovectors via hydrolysis of the ester bond. In contrast, HCNSP with GSH‐cleavable disulfide spacer dramatically suppressed over 95% of IDO‐1 activity of the CT26 tumor cells, which was comparable to that of free NLG919, while 10.7‐fold more efficient than HCNCP, verifying NLG919 released from NLG919‐S‐S‐PPa dimer keeps their IDO‐1 inhibition activity well (Figure S20, Supporting Information).

### 2.4 Pharmacokinetics Profile, Biodistribution and Antitumor Performance of the Prodrug Nanovectors In Vivo

Given the satisfying IDO‐1 inhibition activity of the prodrug nanovectors in vitro, we next sought to investigate the biological behavior of HCNCP and HCNSP in vivo. Balb/c mice were intravenously (i.v.) injected with 100 µL of NLG919, HCNCP or HCNSP at an identical NLG919 dose of 2.6 mg kg^−1^. The serum concentrations of NLG919 and PPa were then detected by HPLC and fluorescence spectrometer measurements, respectively. The prodrug nanovector dramatically elongated the blood circulation and increased the bioavailability of NLG919. For instance, the blood clearance half time (*t*_1/2_β) of the HCNCP and HCNSP groups was 8.1‐fold longer than that of the free NLG919 group, and the bioavailability (area under curves, AUC_0‐t_) of the HCNSP group was ≈17‐fold higher than that of the free NLG919 group, respectively (Figure S21 and Table S1, Supporting information).

CD44 is endogenously expressed in low levels in normal tissues, which is highly expressed in certain types of tumor cells including colorectal cancer.^[^ 44, 45 ^]^ The prodrug nanovectors could passively accumulate at the tumor site through the leaky structure of the tumor‐associated blood vessels. Upon binding with CD44 on the surface of tumor cells, the nanoparticles could be internalized with the tumor cells via CD44–HA interaction. Therefore, HA‐based prodrug nanovectors could achieve tumor‐specific drug delivery by both active and passive tumor targeting properties of the nanoparticles.

**Figure 5:**
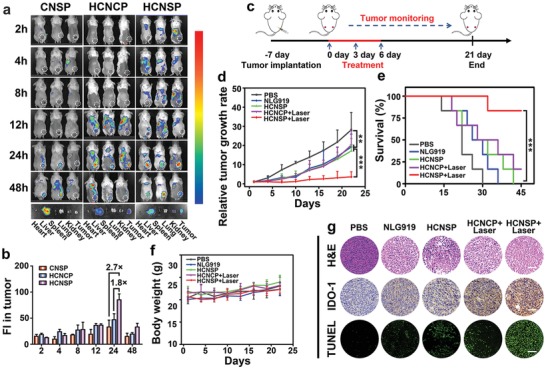
Biodistribution and antitumor performance of the prodrug nanovectors in CT26 tumor‐bearing mice. a) Fluorescence imaging of CNSP, HCNCP, and HCNSP distribution in CT26 tumor‐bearing mice in vivo (Ex = 675 nm, Em = 720 nm). b) Semiquantitative analysis of prodrug nanovectors distribution in the tumors by fluorescence intensity. c) Therapeutic schedule for immune‐photodynamic combination therapy. d) Relative tumor growth curves, e) survival curve and f) the body weight change of CT26 tumor‐bearing mice following the indicated treatments (*n* = 6, mean ± s.d., **p < 0.01, ***p < 0.001). g) H&E staining, TUNEL staining and the immunohistochemical examination of IDO‐1 expression in the tumor sections at the end of the antitumor studies (scale bars = 100 µm).

To investigate the biodistribution of the prodrug nanovectors, CNSP, HCNCP, or HCNSP nanovectors were i.v. injected into CT26 colorectal tumor‐bearing Balb/c mice at an identical dose (5.0 mg kg^−1^) of PPa. The biodistribution of the nanovectors was then examined using fluorescence imaging in vivo at different time points. In comparison with nanoprecipitate of CNSP, HCNCP and HCNSP both displayed obvious tumor accumulation, where the tumoral fluorescence signal was retained up to 48 h postinjection. Interestingly, the fluorescence intensity of HCNSP at the tumor site was 2.7‐ and 1.8‐fold higher than that of CNSP and HCNCP respectively, when examined 24 h postinjection (**Figure** **5**a,b).

We further quantitatively examined the intratumoral distribution of PPa and NLG919 by measuring free NLG919 and PPa concentrations ex vivo by using HPLC and fluorescence spectrometer, respectively. HCNSP group displayed comparable intratumoral PPa concentration while much higher NLG919 distribution in the tumor than that of the HCNCP group, which could be explained by GSH‐induced reduction of the NLG919‐SS‐PPa dimer and release of free NLG919 in the tumor tissues (Figure S22, Supporting information).

The antitumor performance of HCNSP was evaluated in CT26 tumor‐bearing Balb/c mouse model. The tumor‐bearing mice were randomly divided into five groups (*n* = 6) when the tumor volume reached 100 mm^3^. The mice were i.v. injected with NLG919, HCNCP, or HCNSP at an identical dose, i.e., 5.0 and 2.6 mg kg^−1^ of PPa and NLG919, respectively. Then, the tumor was irradiated with 671 nm laser of 200 mW cm^−2^ for 5 min 24 h postinjection. The treatment was repeated three times every 3 d, the tumor volume and body weight were measured during the experimental period (Figure 5c). Monotherapy with free NLG919 or HCNSP moderately reduced tumor growth. PDT with HCNCP (HCNCP+Laser) slightly inhibited tumor growth, whereas photodynamic immunotherapy with HCNSP (HCNSP+Laser) showed the highest antitumor efficacy (Figure 5d). HCNSP+Laser inhibited the growth of CT26 tumor four times more efficient than NLG919, HCNSP or HCNCP+Laser. The superior antitumor performance of HCNSP+Laser combination therapy can be attributed to ICD‐elicited protective immune response and NLG919‐mediated IDO‐1 inhibition. Meanwhile, HCNSP+Laser significantly prolonged the survival rate of tumor‐bearing mice (Figure 5e). No obvious change in the body weight was observed during the treatment (Figure 5f). Hematoxylin‐eosin (H&E) staining of the major organs (i.e., heart, liver, spleen, lung and kidney) indicated negligible histological damage and good biocompatibility of the prodrug nanovectors (Figure S23, Supporting Information).

Posttreatment analysis of the tumor sections by H&E and terminal deoxynucleotidyl transferase dUTP nick‐end labeling (TUNEL) analysis detected significant apoptosis of the tumor cells in the HCNSP+Laser group (Figure 5g). Immunofluorescence staining of the tumor sections posttreatment revealed dramatic ROS generation in the tumor tissue, and CRT expression on the surface of the tumor cells in vivo, and upregulation of IDO‐1 expression in HCNCP+Laser and HCNSP+Laser groups, suggesting IDO‐1‐triggered adaptive immune resistance because of photodynamic immunotherapy in vivo (Figure 5g and Figures S24 and S25, Supporting Information).

### 2.5 Immune Assay and Abscopal Antitumor Effect of the Prodrug Nanovectors In Vivo

**Figure 6:**
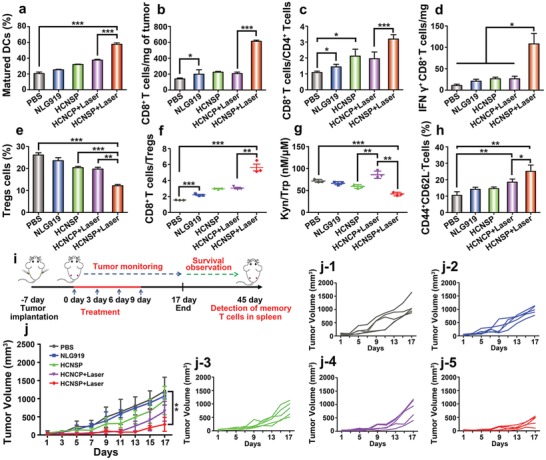
Antitumor performance of prodrug nanovectors. a) DC maturation ratio in the tumor‐draining LNs (***p < 0.001). b) Tumor mass normalized intratumoral infiltration of CD8^+^ T cells. c) CD8^+^T cells to CD4+T cells ratios, d) the number of IFN‐γ^+^ CD8^+^ T cells normalized with tumor mass, e) the frequency of CD4^+^Foxp3^+^ Tregs, and f) the ratio of CD8+ CTLs to Tregs examined 7 d posttreatment by flow cytometric analysis (*n* = 3, mean ± s.d., *p < 0.05, **p < 0.01, ***p < 0.001). g) The intratumoral Kyn to Trp ratio determined using HPLC (*n* = 3, mean ± s.d., **p < 0.01, ***p < 0.001). h) The frequency of memory T lymphocyte in the spleen of CT26 tumor‐bearing Balb/c mice after 30 d treatment (*n* = 3, mean ± s.d., *p < 0.05, **p < 0.01). i) Therapeutic schedule for combination immunotherapy. j) Abscopal tumor growth curves in mice bearing CT26 tumors following the indicated treatments and the specific growth curve of each mouse in each group (*n* = 5, mean ± s.d., **p < 0.01).

To confirm the stimulation of adaptive antitumor immune response, we detected the treatment‐induced DC maturation in vivo. The CT26 tumor‐bearing Balb/c mice were treated by the procedure as described in Figure 5c. HCNCP+Laser induced 1.8‐fold higher DC maturation than that of the PBS group, indicating that PDT‐triggered ICD effect accelerates DC maturation. Owing to the complex two‐way immune feedback, about 1.5‐times higher DC maturation was observed in the HCNSP+Laser group as compared to the HCNCP+Laser group (**Figure** **6**a, Figure S26, Supporting Information).

Matured DCs can present antigens to the naive T lymphocytes to promote the proliferation of CTLs. HCNSP+Laser group significantly promoted intratumoral infiltration of T lymphocytes. Therefore, about three times higher frequency of CD8^+^ T cells in HCNSP+Laser group than HCNCP+Laser group while about 1.5‐times higher ratio of CD8^+^ T cells to CD4^+^ T cells than that of nonreductive response group was observed (Figure 6b,c, Figure S27, Supporting Information). Noticeably, HCNSP+Laser dramatically increased the frequency of IFN‐γ^+^ effector T cells up to 11.9%, which was 1.9‐ and 1.4‐fold more efficient than free NLG919 and HCNCP+Laser, respectively. Furthermore, HCNSP+Laser showed a 3‐fold higher intratumoral infiltration of IFN‐γ^+^ effector T cells than that of the PBS‐treated group (Figure 6d, Figure S28, Supporting Information).

Regulatory T cells (CD4^+^Foxp3^+^CD25^+^ T lymphocytes, namely Tregs) can inhibit the proliferation and activation of CD8^+^ T cells, and therefore the Tregs in intratumoral infiltration are associated with immunosuppressive mechanisms. We thus measured the intratumor infiltration of Tregs in CT26 tumor‐bearing mice. The HCNSP+Laser group showed 12.6% intratumoral infiltration of Tregs, which was 2.2‐times lower than PBS. Moreover, HCNSP+Laser group presented 1.9‐ and 3.2‐fold higher CD8^+^ T cells to Tregs ratio than those of the HCNCP+Laser and PBS group, respectively, implying that codelivery of PPa and active NLG919 tremendously reversed the ITM in the HCNSP+Laser group (Figure 6e,f, Figure S29, Supporting Information). The less NLG919 release from HCNCP was attributed to stable carbon–carbon linkage between NLG919 and PPa.

We further exploited the IDO‐1 inhibition activity of the prodrug nanovectors by determining the Kyn to Trp ratio in the tumor tissue. HCNCP+Laser group showed the highest Kyn to Trp ratio because of laser‐induced secretion of IFN‐γ and increased IDO‐1 expression. However, the subsequent NLG919 release from HCNSP induced a twofold decrease in Kyn to Trp ratio and ultimately contributed to overcoming the immunosuppressive TME (Figure 6g).

Given the robust immune response induced by the synergistic PDT and IDO‐1 inhibition, we next explored whether HCNSP+Laser can induce a systemic immune response to suppress abscopal tumors by using bilateral subcutaneous CT26 tumor‐bearing mice model (Figure 6i). The tumor‐bearing mice were randomly divided into five groups (*n* = 5) and i.v. injected with NLG919, HCNCP, or HCNSP at an identical dose (2.6 mg kg^−1^) of NLG919. The primary tumor was illuminated with 671 nm laser of 200 mW cm^−2^ for 5 min at 24 h postinjection of HCNSP. The treatment was repeated for four times at a time interval of 3 d.

The volume of the abscopal tumors and body weight were measured every 2 d throughout the antitumor study (Figure 6j). Photodynamic immunotherapy and IDO‐1 inhibition by HCNSP+Laser synergistically inhibited the primary tumors. HCNSP+Laser significantly regressed the growth of abscopal tumors, indicating the activation of the systemic antitumor immune response. Negligible body weight loss was observed throughout the antitumor studies, demonstrating good biosafety of the prodrug nanovectors (Figures S30–S32, Supporting Information).

To further explore the mechanism underlying the antimetastasis effect of HCNSP, the effective memory T lymphocytes (T_EM_) in the spleen (CD8^+^CD44^+^CD62L^−^) of CT26 tumor‐bearing Balb/c mice was tested by flow cytometric analysis at 30 d posttreatment. The groups, such as PBS, NLG919, HCNSP, and HCNCP+Laser showed moderate efficacy to generate TEM. In contrast, HCNSP+Laser significantly increased the frequency of TEM in the spleen, which was 2.4‐fold higher than that of the PBS control (Figure 6h, Figure S33, Supporting Information).

NLG919 was clinically tested by oral administration. However, our biodistribution data showed low tumor specificity of NLG919, which might impair the IDO‐1 inhibitory efficacy of NLG919. In contrast, the host–guest prodrug nanovectors administrated through tail vein injection displayed highly improved bioavailability and tumor distribution (Figure S22, Supporting Information). Furthermore, the NLG919‐S‐S‐PPa dimer loaded inside the prodrug nanovectors could be specifically activated inside the tumor cells due to the reduction microenvironment of the tumor cells, which might minimize the side effect of NLG919 in the normal tissues.

The tumor microenvironment is a complex milieu, which comprised of various immunosuppressive pathways and having low immunogenicity. Therefore, it is crucial to simultaneously deliver immune inducer and immune inhibitor for eliciting durable antitumor immunogenicity and downregulating the ITM. The design of the prodrug nanovectors for co‐delivery of PPa (immune inducer) and NLG919 (immune inhibitor) may seem complicated at first sight. However, it is rather a practical design for combination immunotherapy of cancer. The prodrug nanovectors can be readily prepared by one‐step self‐assemble procedure through host–guest interaction between HA‐CD and NLG919‐S‐S‐PPa heterodimer. The resultant prodrug nanovectors displayed tunable drug encapsulation ability and superior colloidal stability to prevent premature drug leakage during the storage and blood circulation. Most importantly, the host–guest interaction‐based self‐assemble strategy could be readily adapted to other combination of immune modulators for immunotherapy of cancer. Taken together, the current study has introduced an easy‐to‐do nanofabrication approach that is practical and cost‐effective for clinical translation.

## 3 Conclusions

In summary, we have developed a supramolecular prodrug nanovector for active tumor targeting and combination immunotherapy of colorectal cancer. The prodrug nanovectors were engineered by host–guest complexation between β‐CD‐grafted HA and reduction‐activatable heterodimer of NLG919‐S‐S‐PPa. The resultant HCNSP nanovectors in combination with 671 nm laser irradiation showed satisfying photoactivity to elicit antitumor immunogenicity by activating ICD cascade. Meanwhile, NLG919‐S‐S‐PPa heterodimer can be cleaved under endogenous GSH conditions to release NLG919 for inactivating IDO‐1 as well as the immunosuppressive Tregs in the tumor microenvironment. Combination photodynamic immunotherapy with the prodrug nanovectors efficiently eradicated both the primary and abscopal tumors by inducing a systemic antitumor immune response. Moreover, the prodrugs demonstrated in this study could be readily adapted to other immune modulators. Owing to the good biocompatibility and biodegradability of HA‐based supramolecular nanovectors, the reported nanoplatform can make great strides in clinical translation of combination immunotherapy.

## 4 Experimental Section

##### Materials and Instruments

Sodium hyaluronic acid (HA, Mw = 36 kDa) was purchased from Bloomage Freda Biopharm Co., Ltd (Shandong, China). Pyropheophorbide a (PPa) and 6‐monodeoxy‐6‐monoamino‐β‐cyclodextrin (NH_2_‐β‐CD, 98%) were purchased from Dibai Chem Tech Co., Ltd (Shanghai, China). NLG919 was purchased from Selleck Chem Co., Ltd (Shanghai, China). Triphosgene and bis(2‐hydroxyethyl) disulfide were purchased from TCI (Shanghai, China). N‐(3‐(dimethylamino)‐propyl)‐N‐ethylcarbodiimide hydrochloride (EDCI), 1‐hydroxybenzotria‐zole anhydrous (HOBT), triethylamine (TEA), 4‐dimethylaminopyridine (DMAP), and trifluoroacetic acid (TFA) were purchased from J&K Scientific Ltd (Beijing, China). Glutathione (GSH), 9,10‐anthracenediyl‐bis(methylene) dimalonic acid (ABDA), 2′,7′‐dichlorofluorescein diacetate (DCFH‐DA) and 3‐(4,5‐dimethylthiazol‐2‐yl)‐2,5‐diphenyltetrazolium bromide (MTT) were all purchased from Sigma‐Aldrich (Shanghai, China). L‐kynurenine (Kyn) and L‐tryptophan (Trp) were all purchased from Dalian Meilun Biotech CO., Ltd (Dalian, China). Regenerated cellulose dialysis bags were purchased from Shanghai Yuanye Bio‐Technology Co., Ltd (Shanghai, China). Sodium dodecyl sulfate (SDS) was purchased from Sinopharm Chemical Reagent Co., Ltd. (Shanghai, China). Hoechst 33342 were obtained from Life Technologies (Shanghai, China). RPMI 1640 medium, fetal bovine serum (FBS), penicillin–streptomycin solution, and trypsin–EDTA solution were all purchased from Gibco (USA). FoxP3 buffer set, anti‐CD11c‐FITC, anti‐CD80‐PE, anti‐CD86‐PE‐Cy7, anti‐CD3‐PerCP‐Cy5.5, anti‐CD4‐FITC, anti‐CD8‐PE, anti‐ForxP3‐PE, anti‐IFN‐γ‐APC, anti‐CD8‐FITC, anti‐CD44‐PerCP‐Cy5.5, and anti‐CD62L‐PE antibodies were all purchased from BioLegend, Inc. (San Diego, USA). Antibodies for calprotectin and indoleamine 2,3‐dioxygenase 1 (IDO‐1) were obtained from Abcam (UK).

The UV–vis absorption spectra were detected by Cary 60 UV–vis spectrophotometer (USA). The fluorescence spectra were measured on Cary Eclipse spectrophotometer (USA). 1H NMR spectra were collected on Bruker Advance spectrometer (400 MHz, Germany). Mass spectrometry was measured on Thermo fisher LTQ Orbitrap Elite. (USA) The dynamic light scattering (DLS) was measured on Malvern Zetasizer Nano ZS (UK). Transmission electron microscopic (TEM) images were observed by JEM‐2100 transmission electronic microscope (Japan). Flow cytometry was examined by FACS Calibur flow cytometric system (UK). The photoactivity was investigated using a 671 nm laser (Changchun New Industries Optoelectronics, China). Fluorescence imaging in vivo was performed by Perkin Elmer Caliper IVIS Lumina II in vivo imaging system (USA). HPLC was acquired with Waters Alliance 2695 HPLC (USA). Confocal laser scanning microscopy (CLSM) was acquired with a Leica TCS‐SP8 confocal scanning microscope (Germany).

##### Cell Lines and Animals

The CT26 colorectal tumor cells were obtained from the cell bank of the Chinese Academy of Sciences (Shanghai, China), and incubated in RPMI 1640 medium with 10% fetal bovine serum and 1% penicillin‐streptomycin at 37 °C in 5% CO_2_ incubator. Four‐week‐old female Balb/c mice were obtained from the Shanghai Experimental Animal Center (Shanghai, China).

##### Synthesis of HA‐CD

HA‐CD was synthesized by conjugating 6‐monodeoxy‐6‐monoamino‐β‐cyclodextrin (NH_2_‐β‐CD) onto hyaluronic acid (HA) via an amide bond. Briefly, HA (41 mg, 0.1 mmol) was dissolved in ultra‐dry DMSO (4 mL), then added with EDCI (57.5 mg, 0.3 mmol) and HOBt (40.5 mg, 0.3 mmol) to activate the carboxyl groups for 2 h. Afterwards, NH_2_‐β‐CD (113.4 mg, 0.1 mmol) and TEA (70 µL, 0.5 mmol) were dissolved in DMSO (2 mL) and slowly added into the HA solution under consistent stirring. The reaction was continued for 24 h at room temperature under N_2_ protection. The reaction product was dialyzed against DI water (MWCO = 14 000 Da) overnight, and lyophilized. HA‐CD was obtained as a white powder (90 mg, yield 58%). The resultant HA‐CD was characterized by 1H NMR spectra.

##### Synthesis of NLG919‐S‐S‐PPa and NLG919‐C‐C‐PPa

NLG919‐SS‐PPa was synthesized by coupling NLG919 (IDO inhibitor) with pyropheophorbide a (PPa) via a disulfide bond. Briefly, triphosgene (252.8 mg, 0.86 mmol), DMAP (908 mg, 7.44 mmol) and NLG919 (600 mg, 2.1 mmol) were dissolved in 5 mL of anhydrous DCM and reacted in argon at room temperature. Then the solution was first concentrated by vacuum evaporation for 1 h, and added 5 mL anhydrous DCM solution of 2,2′‐dithiodiethanol (3199 mg, 8.6 mmol) to the reaction in argon at room temperature. The solution was evaporated and concentrated by vacuum for 1 h to obtain NLG919‐OH. The NLG919‐OH (200 mg, 0.432 mmol), pheophorbide a (PPa), EDCI (95.5 mg, 0.5 mmol) and DMAP (70 mg, 0.57 mmol) (77 mg, 0.144 mmol) were dissolved in 5 mL of DMF and reacted 48 h at room temperature. The solution was evaporated and concentrated by vacuum to obtain the raw product, which was purified by using preparative liquid chromatography. The methanol/water (v/v = 70%/30%, 0.1% TFA) was used as the eluent, and NLG919‐S‐S‐PPa was obtained as a dark green powder (120 mg, yield 28%). The resultant NLG919‐S‐S‐PPa was characterized by ^1^H NMR spectra and mass spectrometry. The reduction insensitive analog of NLG919‐S‐S‐PPa, namely NLG919‐C‐C‐PPa was synthesized by following the same procedure as that of NLG919‐S‐S‐PPa.

##### Preparation of HCNSP and HCNCP

The host–guest prodrug nanovectors were prepared by complexing HA‐CD with NLG919‐S‐S‐PPa through supramolecular interaction. Briefly, HA‐CD (0.0004 g, 0.204 µmol) was dissolved in 1 mL of DI water and NLG919‐S‐S‐PPa (0.0002 g, 0.204 µmol) was dissolved in 150 µL of DMSO, respectively. The DMSO solution of NLG919‐S‐S‐PPa was quickly added into the aqueous solution of HA‐CD under sonication, the mixture was then dialyzed against DI water overnight (MWCO 3500 Da). The resultant prodrug nanovectors were termed as HCNSP. HCNCP was prepared by HA‐CD and NLG919‐C‐C‐PPa, CNSP was composed by CD and NLG‐S‐S‐PPa, NSP was formed by its own self‐polymer. The HCNCP, CNSP, and NSP were prepared by similar procedure. The prodrug nanovectors were stored at 4 °C.

##### Molecular Docking

The molecular docking was performed by Glide in Schrödinger 2015 software package. The molecular structure of β‐CD was obtained from the Protein Preparation Wizard. The hydrogen bonding network of β‐CD was optimized in the OPLS2005 force field to reorient side‐chain hydroxyl groups and mitigate potential steric collides. NLG919 and PPa were drawn from ChemDrawand further optimized by LigPrep panel in Maestro of Schrödinger. In order to forecast the Host‐guest binding tendency, the docking scores were used to illustrate the binding ability.

##### Determination of Binding Stoichiometry by the Continuous Variation Method (Job Plot Method)

The binding stoichiometry of host–guest complex could be determined by Job plot method.^[^ 50 ^]^ In the solution with same volume, keeping the total concentration of host (H) and guest G) but changed the ratio of G to H to prepare a series of solutions. After the solution was determined by ^1^H NMR, the molar fraction X of G (X = [G]/[H]+[G], [G] and [H] represent the concentration of H and G, respectively) was plotted against the chemical shift data. When the host–guest complex of H*_a_*G*_b_* was generated, the ratio of *b* to *a* could be obtained from the maximum point in the curve. The reaction equation of H and G is as follows:

(1) $$aH\;\;+\;\;b\text{G}\;\underset{}{\Leftrightarrow }\;H_{a}{\text{G}}_{b}$$

The *a* and *b* represent the coefficients of the equation. Here, the job plot method was used to determine the binding stoichiometry of NLG919 to β‐CD. The detailed experimental methods were as follows:

Taking β‐CD as the host and NLG919 as the guest to prepare a series of solutions with different ratio of G to H but keep the total concentration at 3.2 × 10^−3^ m. After quiescencing 2 h for ^1^H NMR testing, observing the change of chemical shift of NLG919 and analyzing the data. Draw diagram with [NLG]/[NLG]+[β‐CD] and ∆data∙[NLG] as the abscissa and ordinate respectively and obtain the ratio of b to a (∆data was the chemical shift change of NLG919).

##### Determination of Binding Constant

For the 1:1 host–guest complex system, the equation is as follows:

(2) $$H\;\;+\;\;G\;\overset{K}{\Leftrightarrow }\;HG$$

(3) $$\text{K}\;=\;\;\frac{\left[HG\right]}{\left[H\right]\;\;\times \;\left[G\right]}$$

([H], [G], and [HG] represent the concentration of H, G, and HG, respectively)

Here, the Benese‐Hildebrand equation was used to determine the binding constant between β‐CD with NLG919. In a series of solutions with the same volume, keeping the concentration of NLG919 at 3.5 × 10^−3^ m and increasing the concentration of β‐CD continuously change the ratio of β‐CD to NLG for ^1^H NMR testing. Then, observing the change of chemical shift of β‐CD and analyzing the data. After a series of deformation calculation, the Benese‐Hildebrand equation was obtained as follows:

(4) $$\frac{1}{\Delta }\;\;=\;\;\frac{1}{\Delta_{0}\cdot K\cdot \left[H\right]}\;+\;\;\frac{1}{\Delta_{0}}$$

(∆ was the value of chemical shift change between free state and binding equilibrium state of H (β‐CD); ∆_0_ was the value of chemical shift change between free state and 1:1 binding equilibrium state of H (β‐CD)).

After formatting the equation again, the final equation could be obtained as follows

(5) $$\frac{\Delta }{\left[H\right]}\;\;=\;-\Delta K\;\;+\;\;\Delta_{0}K$$

Draw diagram with ∆ and ∆/[H] as the abscissa and ordinate respectively and obtain the slope *K*.

##### Characterization of the Prodrug Nanovectors

The hydrodynamic diameter, size polydispersity (PDI), and surface ζ – potential of the HCNSP, HCNCP, CNSP, and NSP prodrug nanovectors were examined using dynamic light scattering (DLS) measurement. The serum stability of the prodrug nanovectors was examined in 10 (v/v)% of FBS by using DLS, UV–vis spectroscopy and fluorescence spectroscopy (Ex = 415 nm, Em = 500–800 nm) at the desired time durations (3, 6, 9, 12 h). The photoactivity of the prodrug nanovectors was evaluated by fluorescence probe‐ABDA using UV–vis spectroscopy.

The reduction‐sensitivity of the prodrug nanovectors was evaluated by measuring GSH‐induced NLG919 release using HPLC. Briefly, the prodrug nanovectors were incubated in PBS with 10.0 × 10^−3^ m GSH at 37 °C. Then the solution was examined using HPLC at 0.5, 1, 2, 4, 8, 12, and 24 h with methanol and water (0.1% trifluoroacetic acid) (v/v = 95%/5%) at 0.5 mL min^−1^.

##### Phototoxicity of the Prodrug Nanovectors In Vitro

To examine the cytotoxicity of the HCNSP and HCNCP prodrug nanovectors, CT26 cell were seeded in 96‐well tissue culture plates at a density of 3000 cells per well and incubated with HCNSP, HCNCP at series of concentrations for 24 h. Then the cell viability was detected by MTT assay.

To determine the phototoxicity of the prodrug nanovectors, CT26 cell were seeded in 96‐well tissue culture plates at a density of 3000 cells per well and incubated with HCNSP at an identical PPa concentration of 1.0 × 10^−6^ m. After 24 h, washing twice with PBS and replacing the fresh cell culture medium. Then the cells were irradiated with a 670 nm laser for 30 s at photodensity of 100, 200, or 400 mW cm^−2^. The cells were cultured for further 24 h and then detected by MTT assay.

##### Cellular Uptake of Prodrug Nanovectors In Vitro

To analyze the intracellular uptake of the prodrug nanovectors, the cells were seeded in 24‐well tissue culture plates (30 000 cells per well) and incubated with different HCNSP at a PPa concentration of 1.0 × 10^−6^ m for 1, 2, 3, 4 h, respectively. Next the cells were trypsinized, resuspended in PBS, and examined by flow cytometric measurement.

To investigate the intracellular distribution of the prodrug nanovectors, CT26 cell were seeded on 25 mm glass bottom dishes (30 000 cells per well). After 24 h preincubation, the cells were incubated with various nanovectors at a PPa concentration of 1 × 10^−6^ m for 4 h. Then the cells were stained with Hoechst 33342, washed twice with PBS, fixed with 4% paraformaldehyde and imaged with CLSM.

##### ROS Generation In Vitro and In Vivo

To detect PDT‐induced ROS generation, CT26 cells seeded in six‐well tissue culture plates (1.0 × 10^5^ cells per well) were incubated with HCNSP at a PPa concentration of 1 × 10^−6^ m for 4 h. Afterwards, the cells were incubated with 10 × 10^−6^ m of fluorescent probe DCFH‐DA for 20 min, and washed with PBS, harvested, resuspended in 50 µL PBS. The tumor cells were then irradiated with 671 nm laser for 5 min at photodensity of 100, 200, or 400 mW cm^−2^ and the intracellular fluorescence intensity of DCF was quantitative examined by flow cytometric measurement.

To visualize PDT‐induced ROS generation in vitro, CT26 cells were seeded in 25 mm glass bottom dishes (30 000 cells per well) and incubated with HCNSP for 4 h at a PPa concentration of 1 × 10^−6^ m. After stained with Hoechst 33342 and DCFH‐DA for the appropriate time, the cells were washed, fixed, irradiated with a 671 nm laser at a photodensity of 200 mW cm^−2^ for 2 min, and imaged by CLSM.

Laser‐induced intratumoral ROS generation was examined by CLSM examination ex vivo. When the tumor volume reached 200–300 mm^3^, the mice were intravenously (i.v.) injected with HCNSP micelle at a PPa dose of 5.0 mg kg^−1^. Twenty‐four h later, the mice were intratumorally injected with DCFH‐DA at a dose of 2.5 mg kg^−1^. Thirty minutes later, the tumor was irradiated with a 671 nm laser for 5 min at a photodensity of 400 mW cm^−2^. Finally, the tumor was collected, frozen sectioned at 5.0 µm thickness, stained with DAPI and observed by CLSM.

##### Immunogenic Cell Death Induction of Prodrug Nanovectors In Vitro

To investigate treatment‐induced surface expression of CRT on the tumor cells and extracellular release of HMGB1, CT26 cells were seeded in the 24‐well tissue culture plate (30 000 cells per well). After 24 h of incubation, the cells were incubated with prodrug nanovectors or NLG919 at an identical PPa or NLG919 concentration of 0.5 m for 12 h. Then the cells were washed twice and irradiated with laser at a photodensity of 50 mW cm^−2^ for 30 s in HCNSP or HCNCP with laser group. After 4 h of incubation, the cells were washed with PBS twice and fixed with 0.25% paraformaldehyde for 5 min. After washing with PBS twice, the cells were first stained with primary antibody for 30 min and next stained with Alexa488‐conjugated monoclonal secondary antibody for another 30 min. Finally, the cells were washed with PBS twice and analyzed by flow cytometric measurement.

Extracellular HMGB1 release was examined using immunofluorescence analysis. Briefly, CT26 tumor cells were seeded on a live cell imaging glass bottom dish at a density of 2 × 10^4^ cells per well for 24 h. The cells were then incubated with free NLG919, HCNSP, or HCNCP for 12 h at an identical PPa concentration of 0.5 × 10^−6^ m. The HCNSP and HCNCP groups were then illuminated with 671 nm laser for 30s at photodensity of 50 mW cm^−2^. The cells were cultured for additional 12 h, and stained with anti‐HMGB1 antibody. The cells were stained with DAPI and examined by CLSM.

To visually treatment‐induced surface expression of CRT on the tumor cells, immunofluorescence analysis was used to verify the immunogenic cell death of cells. First, CT26 cells were seeded on 25 mm glass bottom dishes (30 000 cells per well) for 24 h and then treated with prodrug nanovectors or NLG919 at an identical PPa or NLG919 concentration of 0.5 m for 12 h. The cells were then washed twice and illuminated with 671 nm laser for 30 s at photodensity of 50 mW cm^−2^. After 4 h of incubation, the cells were washed with PBS twice and fixed with 4% paraformaldehyde for 20 min. After washing with PBS twice, the cells were first stained with primary antibody for 1 h and next stained with Alexa 488‐conjugated monoclonal secondary antibody for another 30 min. Finally, the cells were washed, stained with DAPI and imaged by CLSM.

##### IDO‐1 Inhibitory Effect of the Prodrug Nanovectors In Vitro

To exploit the IDO‐1 inhibitory activity of the prodrug nanovectors in vitro, 5.0 × 10^4^ CT26 tumor cells were seeded in the six‐well plate for 24 h. The cells were then treated with 100 ng mL^−1^ of IFN‐ɤ for 24 h. Afterwards, the cells were further incubated with free NLG919, HCNCP, or HCNSP for 36 h at an identical NLG919 concentration of 5.0 µg mL^−1^. The cells were then harvested, lysized, and lyophilized. The cell lysis was then dispersed in 30% aqueous solution of trichloroacetic acid to precipitate the protein. The supernatant was collected by centrifugation for examining kynurenine and tryptophan concentration using HPLC.

##### DC Maturation In Vitro

To study the DC maturation in vitro, BMDCs were extracted from the bone marrow of 8‐week old Balb/c mice. The immature DC cells were cocultured with corresponding drug pretreated CT26 cells for 24 h and then analyzed by flow cytometry after staining with anti‐CD11c‐FITC, anti‐CD80‐PE, anti‐CD86‐PE‐Cy7 antibodies.

##### Pharmacokinetics Profile of the Prodrug Nanovectors In Vivo

To test the pharmacokinetics profile of NLG919, HCNCP, and HCNSP, Balb/c mice (*n* = 3) were intravenously injected with 100 µL of NLG919, HCNCP, or HCNSP suspension respectively, at an identical NLG919 dose of 2.6 mg kg^−1^ and PPa dose of 5.0 mg kg^−1^, respectively. The blood samples were then collected at 5 min, 15 min, 1 h, 2 h, 4 h, 8 h, 16 h, 24 h and 48 h postinjection. The concentration of NLG919 and PPa was detected by HPLC and fluorescence photospectrometer, respectively.

##### Biodistribution of the Prodrug Nanovectors In Vivo

To examine biodistribution of the prodrug nanovectors in vivo, 2 × 10^6^ CT26 tumor cells were subcutaneously injected into the right mammary gland of Balb/c mice. The mice were randomly grouped (*n* = 3) when the tumor volume reached 100 mm^3^. The mouse groups were orally administrated with 100 µL of NLG919, or intravenously injected with 100 µL of NLG919, CNSP, HCNCP or HCNSP suspension at an identical NLG919 dose of 2.6 mg kg^−1^ or PPa dose of 5.0 mg kg^−1^, respectively. The whole body fluorescence imaging were obtained at 2, 4, 8, 12, 24, and 48 h pi using Caliper IVIS Lumina II in vivo imaging system. Then the mice were sacrificed at 8, 24, and 48 h after nanovectors administration. The main organs and tumor were collected and examined using fluorescence imaging ex vivo. The mice were then sacrificed at 1, 2, 4, 8, 24, and 48 h to collect the tumors. The tumors were homogenized and were dissolved in methanol. NLG919 concentrations in the supernatant were then examined by HPLC measurement. PPa concentrations in the supernatant were examined by fluorescence spectrometer.

##### Antitumor Effect and Biosafety Assay In Vivo

To investigate the antitumor effect of the prodrug nanovectors in vivo, Balb/c mice were implanted with CT26 tumor on the right side between the tail and thigh. The mice were randomly grouped when the tumor volume reached 100 mm^3^ (PBS, NLG919, HCNSP, HCNCP+Laser and HCNSP+Laser). The mice were i.v. injected with NLG919, HCNCP or HCNSP at an identical PPa dose of 5.0 mg kg^−1^ and NLG919 dose of 2.6 mg kg^−1^, respectively. Twenty four hours pi, the tumors in HCNCP+Laser and HCNSP+Laser groups were locally irradiated with a 671 nm laser at photodensity of 200 mW cm^−2^ for 5 min. The treatment was repeated for three times at a time interval of 3 d. The body weight and tumor volume of the mice were measured every three days for a total of 21 d. The tumor volume was calculated by the formula:

(6) $$V\;=\;\;\left(L\;\;\times \;\;W\;\;\times \;\;W\right)\text{/}2$$

(*L*, the longest dimension; *W*, the shortest dimension) and expressed by the relative tumor growth rate by normalizing with the initial tumor volume. According to the protocol of the animal study, animal death was recorded when the tumor volume reached 2000 mm^3^. To assess the biosafety, the major organs (heart, liver, spleen, lung, kidney) and tumor were examined by hematoxylin‐eosin (H&E) staining.

##### Abscopal Antitumor Effect In Vivo

The immune‐photodynamic combined anticancer efficacy at the abscopal was tested in CT26 tumor‐bearing mice. 1 × 10^6^ CT26 were s.c. injected into the right flank and 5 × 10^5^ CT26 cells were s.c. injected into the left flank to obtain the bilateral tumor‐bearing mice. The mice were randomly divided into five groups (*n* = 5) when the tumor volume reached 100 mm^3^ and accepted treatments only on the right‐sided tumors. The treatment was repeated for four times at a time interval of 3 d. The body weight and two side tumor volume of the mice were measured every two days for a total of 17 d to study the abscopal antitumor effect.

##### Kynurenine and Tryptophan Measurement In Vivo

To determine Kyn and Trp in the tumor, the tumor tissues were harvested from the Balb/c tumor bearing mice with different treatment. The tissue was homogenized with homogenizer, and the homogenate was dissolved in 10% trichloroacetic acid to precipitate the proteins. Finally, the concentration of Kyn and Trp in the supernatant was detected by HPLC.

##### DC Maturation In Vivo

To investigate DC maturation in vivo, lymph nodes of mice were generated from Balb/c tumor bearing mice with different treatment and grinded to obtain lymphocyte single cell suspension using a syringe piston. After staining with anti‐CD11c‐FITC, anti‐CD80‐PE, anti‐CD86‐PE‐Cy7 antibody according to the manufacturer's protocols, DC cells were analyzed using flow cytometry.

##### T Lymphocytes Infiltration in Tumor

To detect the T lymphocytes infiltration in tumor, the tumors were collected, cut into small pieces and homogenized. Then the homogenate were immersed at 37 °C for 45 min in the solution of 1 mg mL^−1^ collagenase IV and 0.2 mg mL^−1^ DNase I. After dissociation, single cell suspension was obtained by filtration with membrane. And lymphocyte isolates were then used to obtain lymphocyte from single cell suspension solution. According to the instructions, the single cells were finally stained with anti‐CD3‐PerCP‐Cy5.5, anti‐CD4‐FITC, anti‐CD8‐PE and anti‐IFN‐γ‐APC antibodies to analyze the CTLs (CD3^+^CD4^−^CD8^+^) and CD4+ T cells (CD3^+^CD4^+^CD8^−^), and measured by flow cytometry. To analyze the Tregs (CD3^+^CD4^+^Foxp3^+^), the lymphocytes were stained with anti‐CD3‐PerCP‐Cy5.5, anti‐CD4‐FITC, anti‐Foxp3‐PE antibodies and measured by flow cytometry.

##### Immune Memory Effect of CD8^+^ T Lymphocytes in the Spleen

To examine the memory T lymphocytes in spleens, the spleen was gently pressed with the piston of syringe, and the single cell suspension was obtained by filtering membrane. Then the single cells were stained with anti‐CD8‐FITC, anti‐CD44‐PerCP‐Cy5.5 and anti‐CD62L‐PE antibodies according to the instructions to analyze the TEM (CD8^+^CD44^+^CD62L^−^).

##### Statistical Analysis

Results are given as mean ± SD. One‐way analysis of variance (ANOVA) was used to determine the significance of the difference. Statistical significance was set at *p < 0.05, **p < 0.01, ***p < 0.001.

## Conflict of Interest

The authors declare no conflict of interest.

## Supporting information

Supporting Information
