# Supplementary material for: Supramolecular Prodrug Nanovectors for Active Tumor Targeting and Combination Immunotherapy of Colorectal Cancer
Source: Adv Sci (Weinh). 2020 Feb 25;7(8):1903332. doi: 10.1002/advs.201903332 (PMC7175291; doi:10.1002/advs.201903332)
Supplement: Supplementary file 1 — Supporting Information [file ADVS-7-1903332-s001.pdf]

Copyright WILEY-VCH Verlag GmbH & Co. KGaA, 69469 Weinheim, Germany, 2020.

ADVANCED SCIENCE
Open Access

Supporting Information

for *Adv. Sci.,* DOI: 10.1002/advs.201903332

Supramolecular Prodrug Nanovectors for Active Tumor Targeting and Combination Immunotherapy of Colorectal Cancer

*Xianli Hu, Bo Hou, Zhiai Xu, Madiha Saeed, Fang Sun, Zhenmei Gao, Yi Lai, Tong Zhu, Fan Zhang, Wen Zhang, and Haijun Yu**

Supporting Information

For

Supramolecular Prodrug Nanovectors for Active Tumor Targeting and Combination Immunotherapy of Colorectal Cancer

Xianli Hu, Bo Hou, Zhiai Xu, Madiha Saeed, Fang Sun, Zhenmei Gao, Yi Lai, Tong Zhu, Fan Zhang, Wen Zhang, Haijun Yu*

X. Hu, B. Hou, Prof. Z. Xu, Z. Gao, Y. Lai, Prof. T. Zhu, Prof. W. Zhang

School of Chemistry and Molecular Engineering

East China Normal University

Shanghai 200241, China

X. Hu, B. Hou, Dr. M. Saeed, F Sun, Prof. H. Yu

State Key Laboratory of Drug Research & Center of Pharmaceutics

Shanghai Institute of Materia Medica, Chinese Academy of Sciences

Shanghai 201203, China

E-mail: hjyu@simm.ac.cn

Prof. F. Zhang

Department of Chemistry

Fudan University

Shanghai, 200438, China

Table of Contents

Table S1. Page 4

Figure S1. Page 5

Figure S2. Page 6

Figure S3. Page 7

Figure S4. Page 8

Figure S5. Page 9

Figure S6. Page 10

Figure S7. Page 11

Figure S8. Page 12

Figure S9. Page 13

Figure S10. Page 14

Figure S11. Page 15

Figure S12. Page 16

Figure S13. Page 17

Figure S14. Page 18

Figure S15. Page 19

Figure S16. Page 20

Figure S17. Page 21

Figure S18. Page 22

Figure S19. Page 23

Figure S20. Page 24

Figure S21. Page 25

Figure S22. Page 26

Figure S23. Page 27

Figure S24. Page 28

Figure S25. Page 29

Figure S26. Page 30

Figure S27. Page 31

Figure S28. Page 32

**Figure S29.**	Page 33
**Figure S30.**	Page 34
**Figure S31.**	Page 35
**Figure S32.**	Page 36
**Figure S33.**	Page 37

Table S1. Pharmacokinetic parameters of free NLG919, HCNCP and HCNSP examined by determining the blood concentration of NLG919 in vivo.

	**NLG919**	**HCNCP**	**HCNSP**
**$t_{1/2}\beta$ (h)**	0.79±0.06	6.46±0.91	6.67±1.64
**C_{max} (μg/mL)**	2.79±0.25	5.29±0.28	6.03±0.36
**CL (L/h/kg)**	1.13±0.010	0.087±0.006	0.078±0.008
**AUC_{0-t} (mg/L*h)**	2.08±0.10	27.28±2.47	35.78±3.71

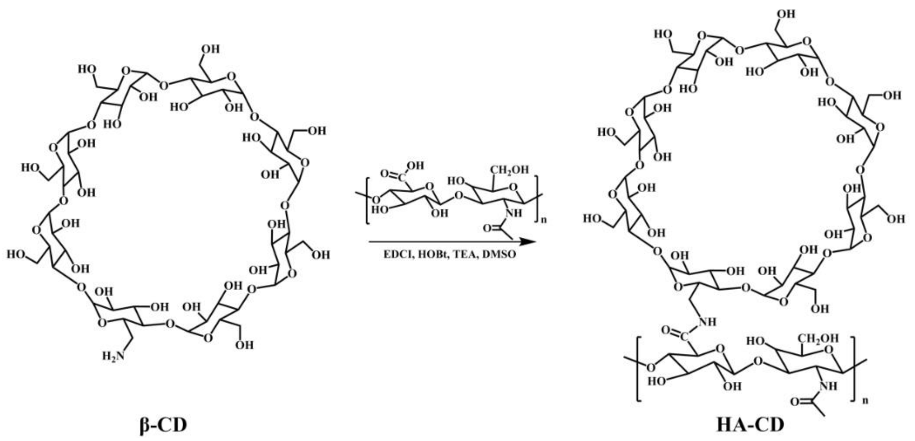

Figure S1. Schematic illustration for the synthesis of HA-CD.

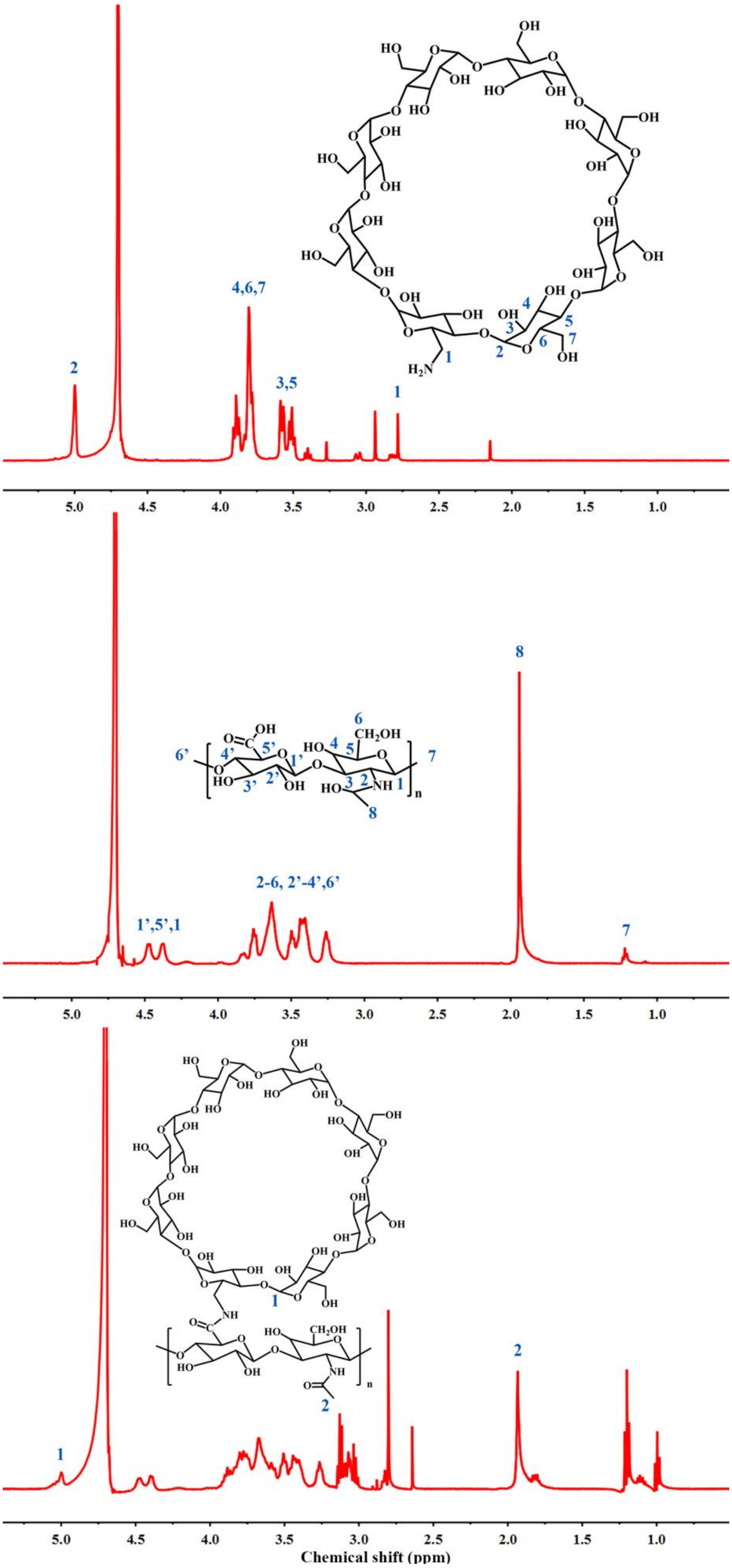

Figure S2. ^{1}H-NMR spectra of HA-CD (a) HA-CD (b) CD (c) HA.

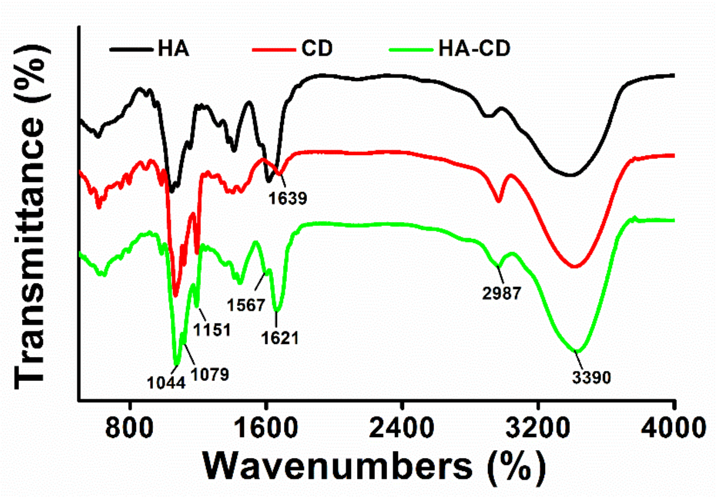

Figure S3. FTIR spectra of HA, NH_2-β-CD and HA-CD, respectively.

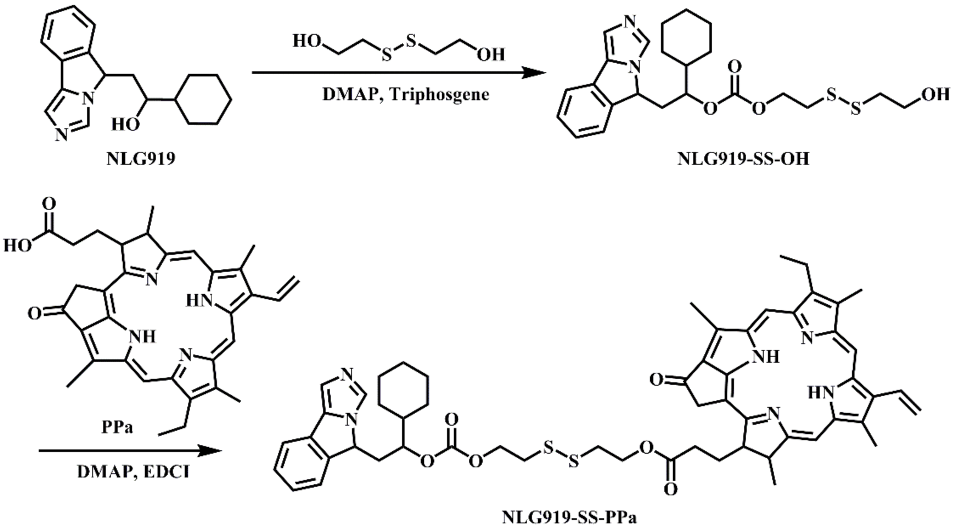

Figure S4. The synthetic route of NLG919-S-S-PPa dimer.

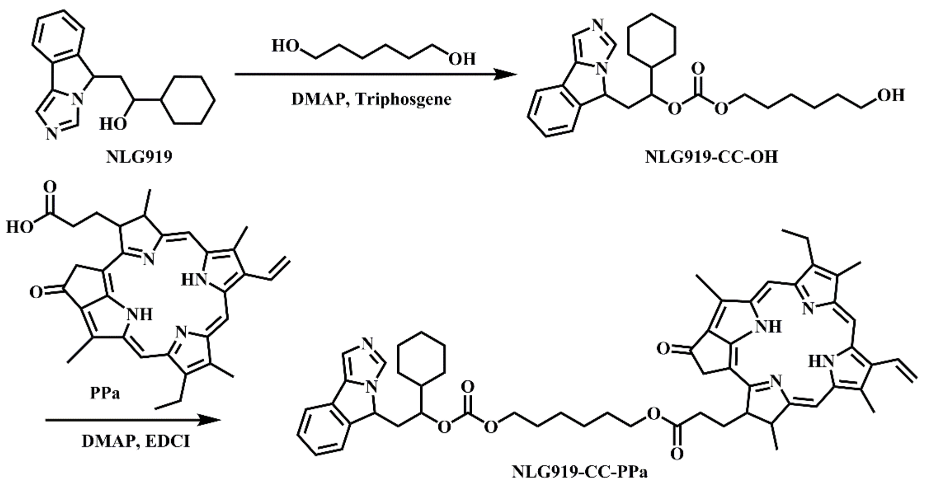

Figure S5. The synthetic route of NLG919-C-C-PPa dimer.

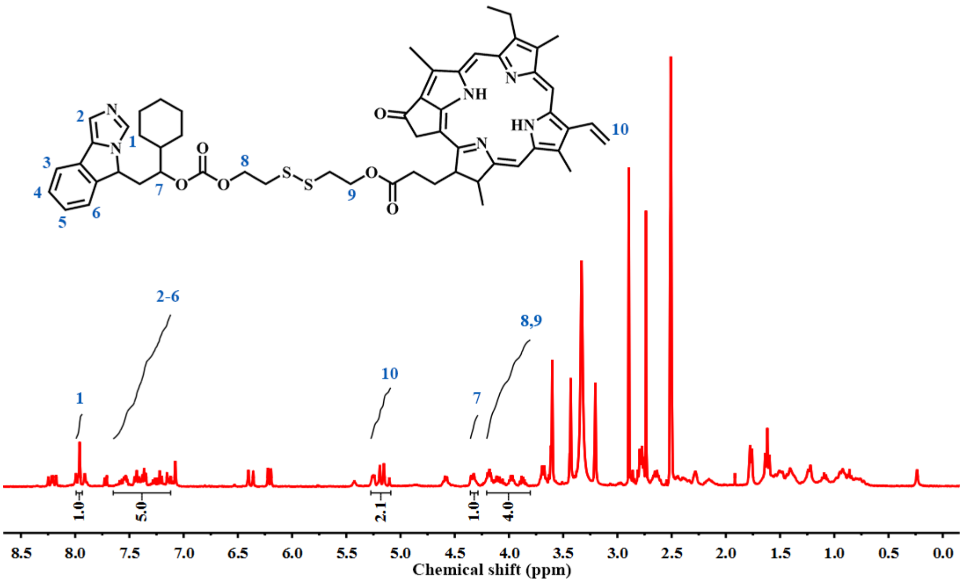

Figure S6. The ^{1}H-NMR spectrum of NLG919-S-S-PPa.

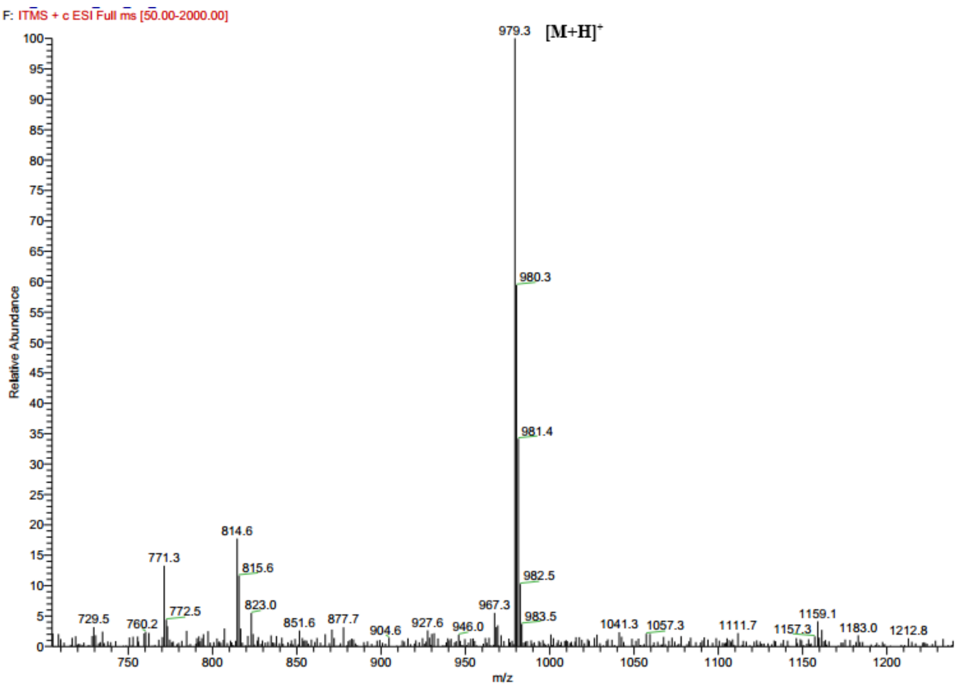

Figure S7. The ESI-MS spectrum of NLG919-S-S-PPa.

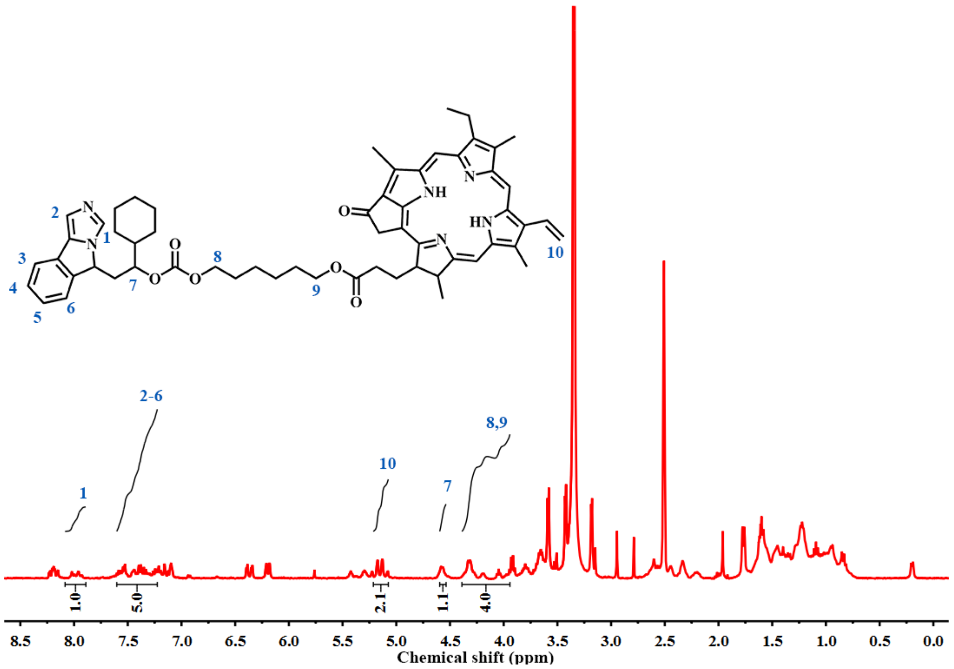

Figure S8. The representative ^{1}H-NMR spectrum of NLG919-C-C-PPa dimer.

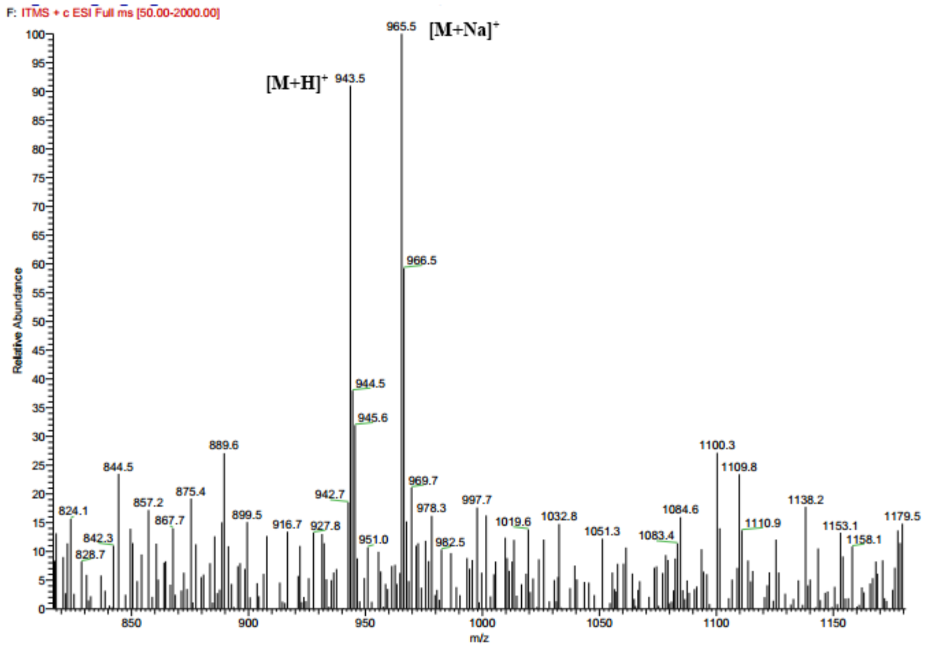

Figure S9. The ESI-MS spectrum of NLG919-C-C-PPa.

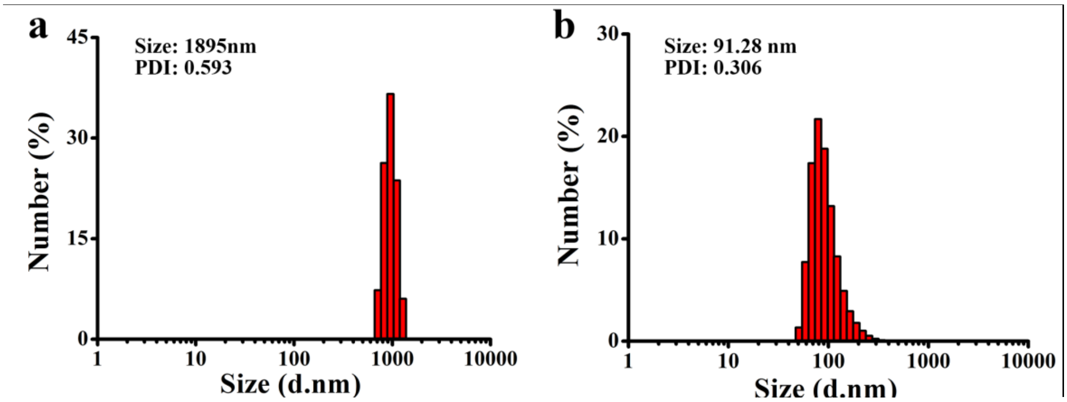

Figure S10. Hydrodynamic diameter of CNSP(a) and NSP(b).

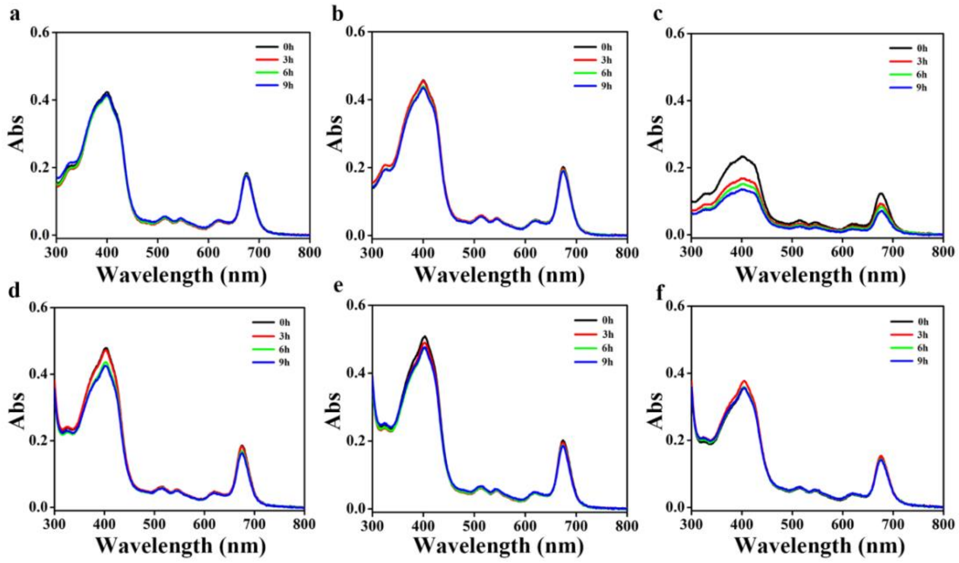

Figure S11. UV-Vis spectroscopic examination of the stability of HCNSP, HCNCP and CNSP nanovectors, respectively. (a)-(c) was the stability of HCNSP, HCNCP and CNSP in PBS without 10% serum addition at 37 ℃ for the desired time duration. (d)-(f) was the stability of HCNSP, HCNCP and CNSP incubated in PBS with 10% serum addition at 37 ℃ for the desired time duration.

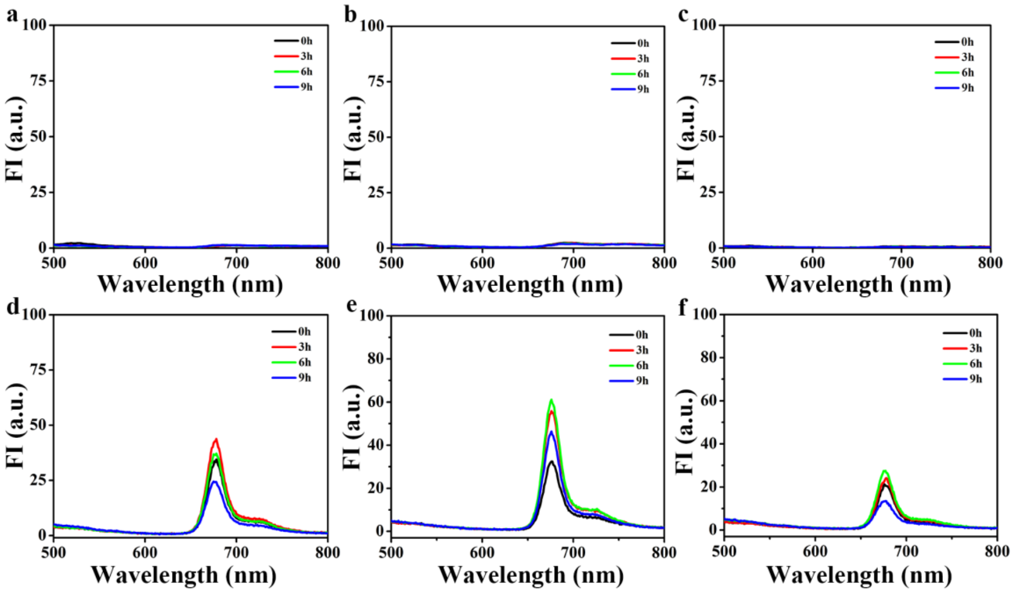

Figure S12. Fluorescence spectroscopic examination of the stability of HCNSP, HCNCP and CNSP nanovectors, respectively. (a)-(c) was the stability of HCNSP, HCNCP and CNSP in PBS without 10% serum addition at 37 ℃ for the desired time duration. (d)-(f) was the stability of HCNSP, HCNCP and CNSP incubated in PBS with 10% serum addition at 37 ℃ for the desired time duration.

.

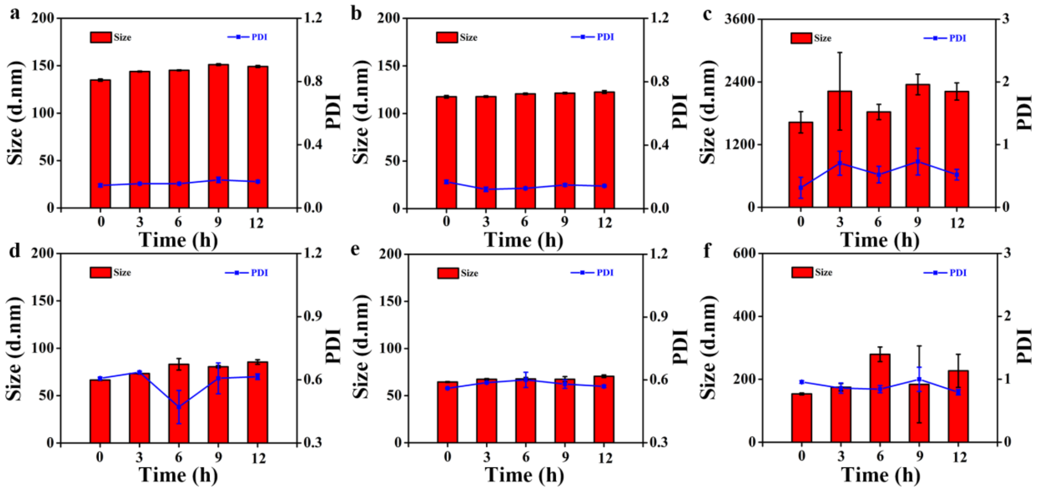

Figure S13. DLS examination of the stability of HCNSP, HCNCP and CNSP nanovectors, respectively. (a)-(c) was the stability of HCNSP, HCNCP and CNSP in PBS without 10% serum addition at 37 ℃ for the desired time duration. (d)-(f) was the stability of HCNSP, HCNCP and CNSP incubated in PBS with 10% serum addition at 37 ℃ for the desired time duration.

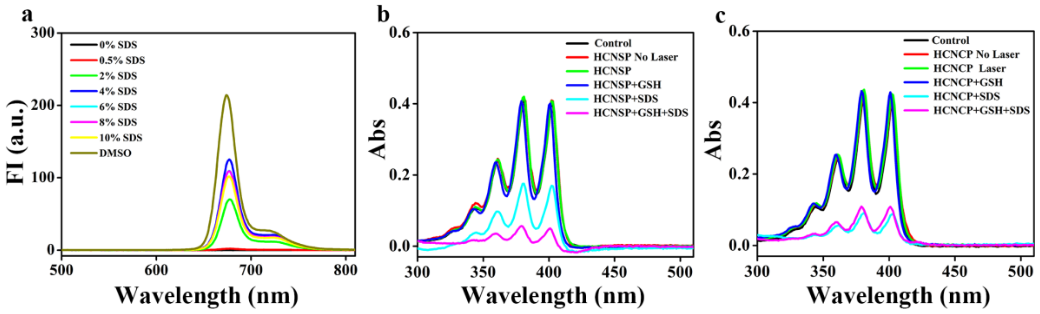

Figure S14. Fluorescence and photoactivity of HCNCP and HCNSP nanovectors, respectively. (a) Fluorescence recovery at different SDS concentrations. Fluorescence recovery of HCNSP (b) and HCNCP (c) under GSH, SDS or both GSH and SDS conditions.

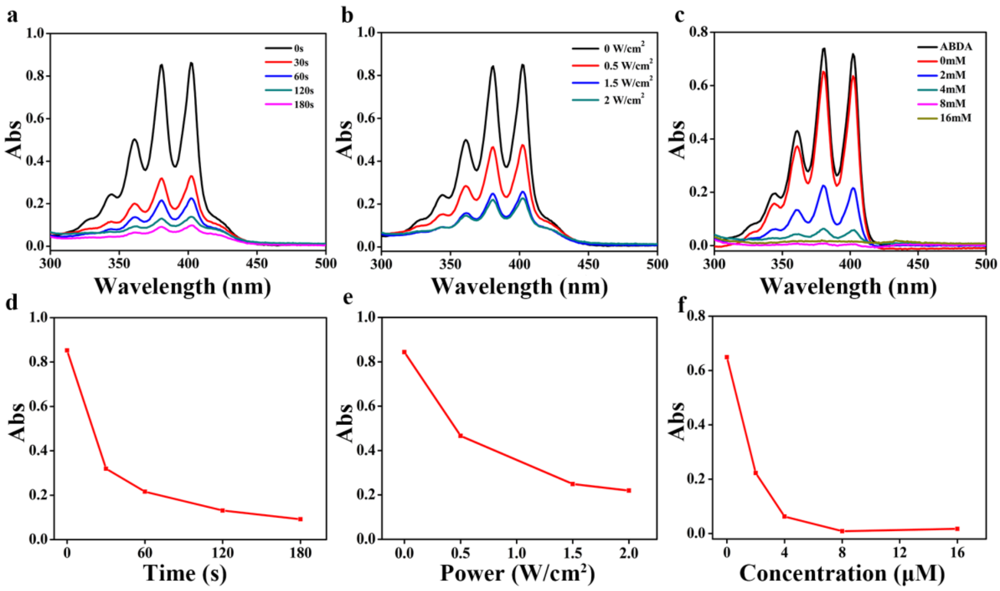

Figure S15. The dependence of prodrug nanovectors photodynamic effects on the irradiation time (a,d), photodensity (b,e) and nanovector concentration (c,f).

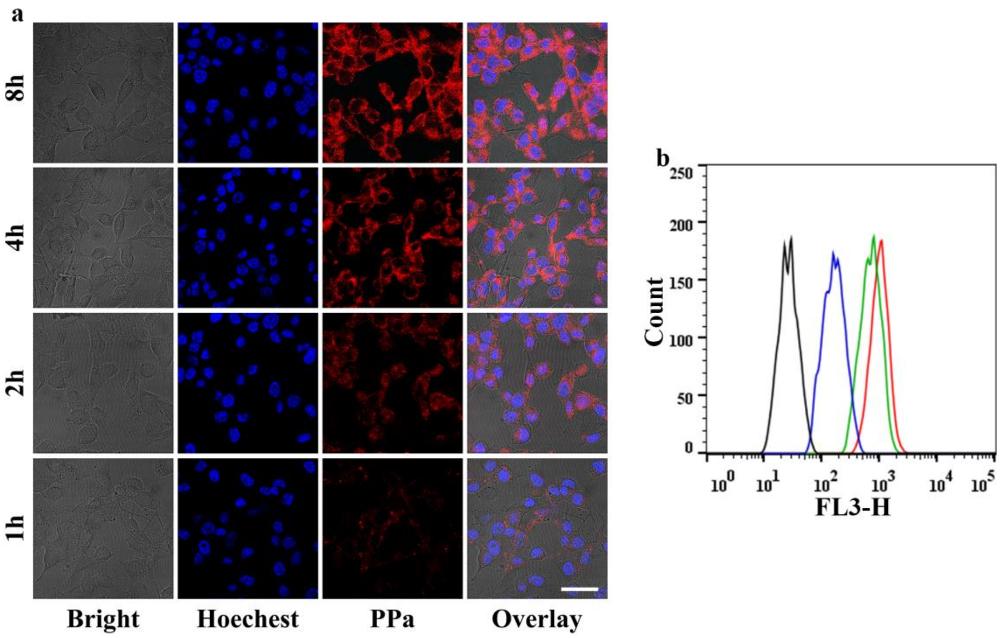

Figure S16. CLSM (a) and flow cytometric examination (b) of time-dependent intracellular uptake of HCNSP in CT26 cells (Scale bar =50 μm).

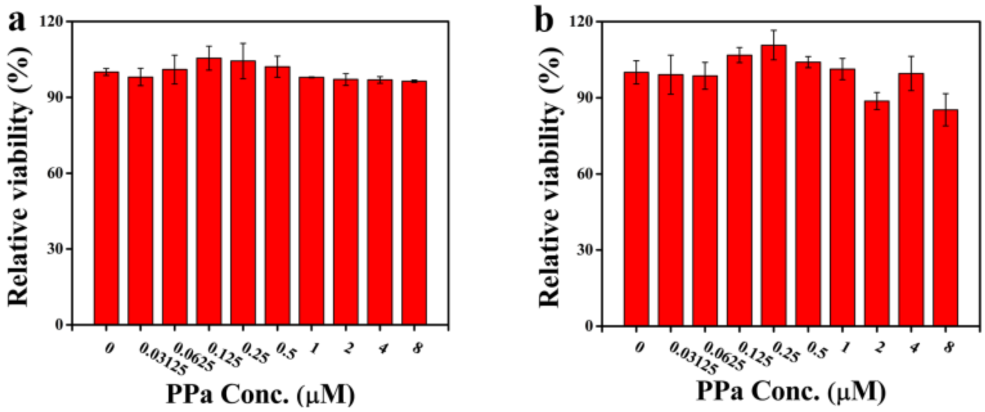

Figure S17. Cytotoxicity assay of HCNSP (a) and HCNCP (b) nanovectors in CT26 cells, respectively.

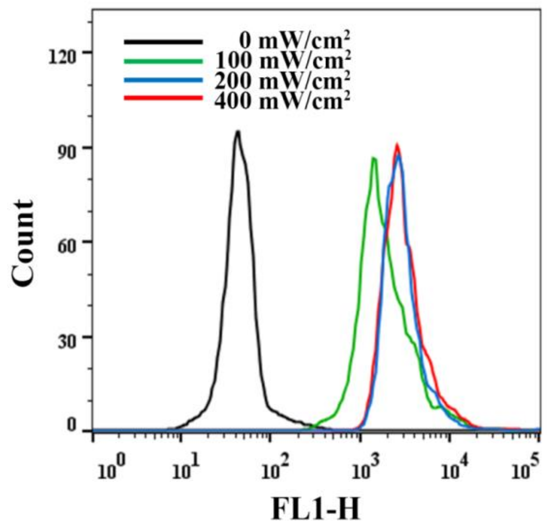

Figure S18. Flow cytometric analysis of PDT-induced ROS generation in CT26 cells in photodensity-dependent manner.

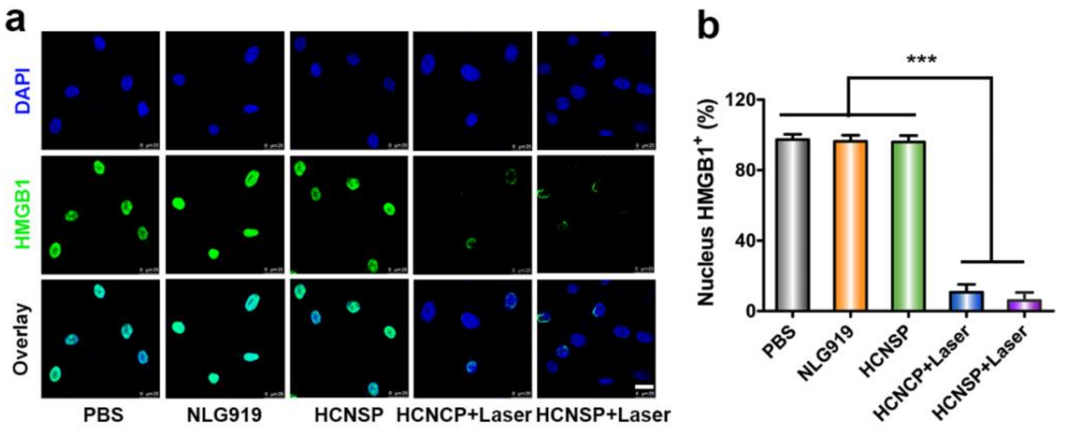

Figure S19. (a) The representative CLSM images of PDT-triggered HMGB1 release in CT26 cells (scale bar = 25 μm); (b) The semi-quantitative analysis of HMGB1 release in CT26 cells.

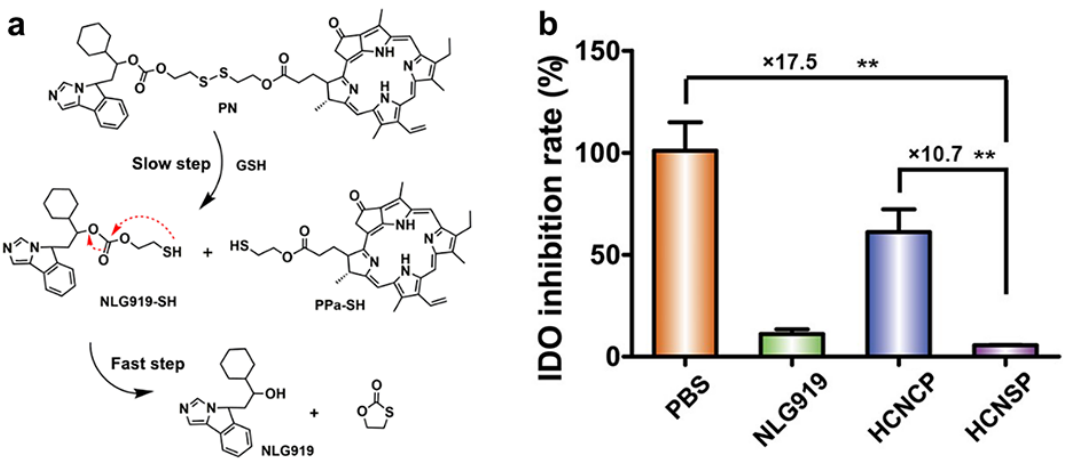

Figure S20. (a) Reaction mechanism for GSH-triggered reduction of NLG919-SS-PPa dimer; (b) IDO-1 inhibition activity of free NLG919, HCNCP and HCNSP in CT26 tumor cells in vitro. The IDO-1 activity was determined by measuring the intracellular concentrations of Trp and Kyn.

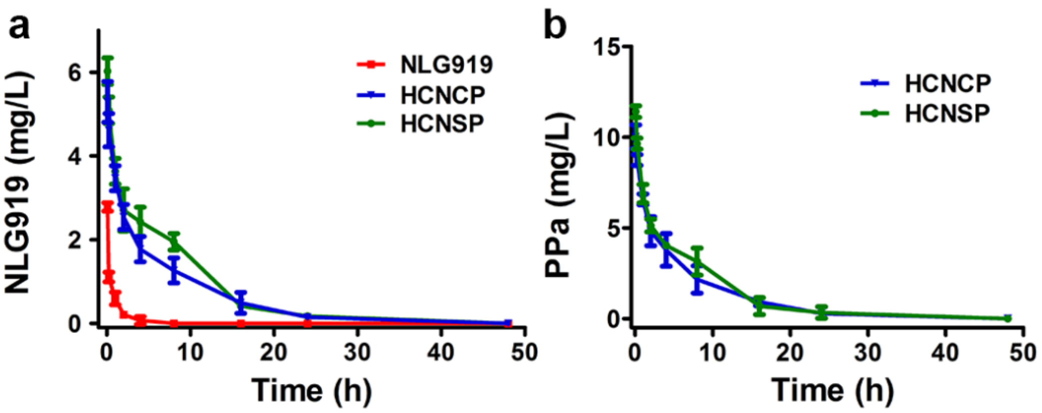

Figure S21. Pharmacokinetics profile of free NLG919, HCNCP and HCNSP examined as a function of blood concentration of NLG919 and PPa, respectively.

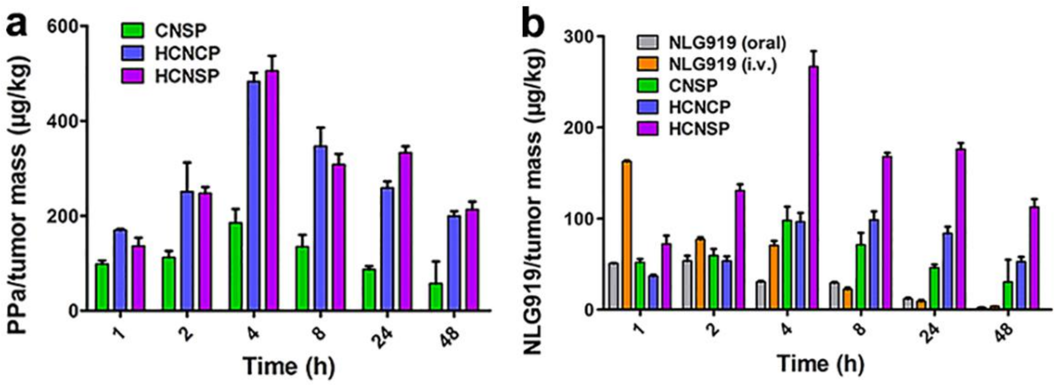

Figure S22. Intratumoral PPa and NLG919 distribution of free NLG919, CNSP, HCNCP and HCNSP, respectively. PPa and NLG919 were both extracted from the tumor tissue. The intratumoral PPa and NLG919 concentrations were then examined by fluorescence spectrometer and HPLC measurement, respectively.

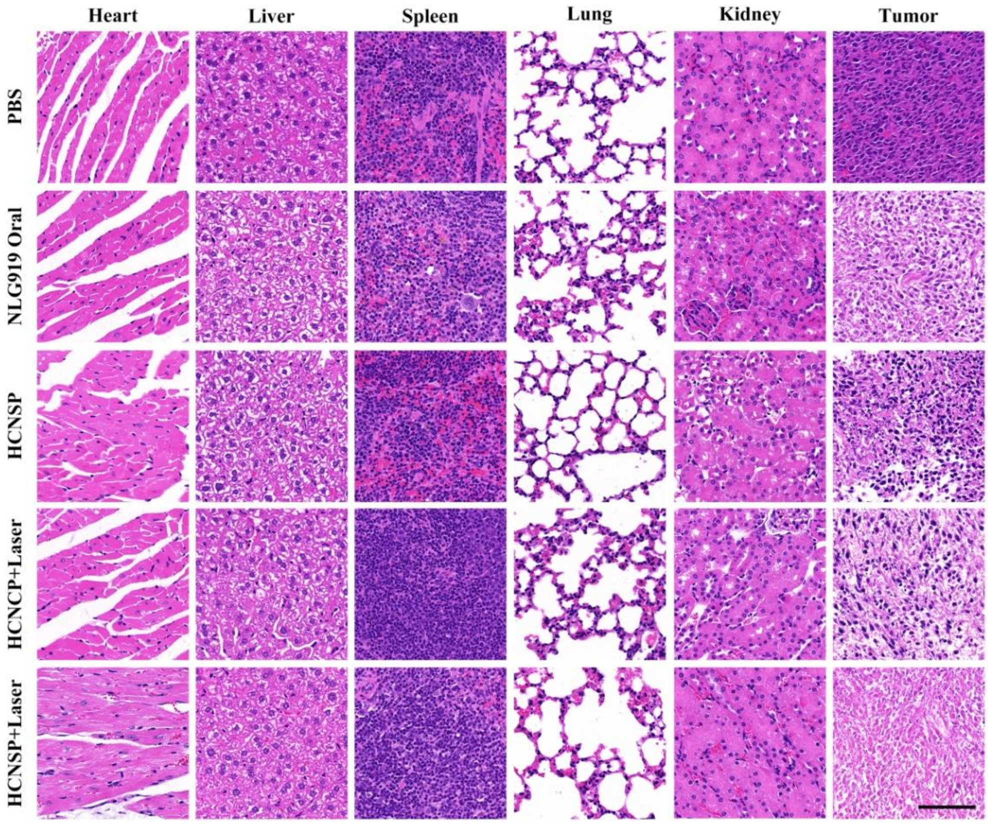

Figure S23 Histochemical examination of the heart, liver, spleen, lung, kidney and tumors. All the major organs (heart, liver, spleen and kidney) were harvested at the end of the antitumor study and fixed in 10% formalin solution, dehydrated, sliced into 5.0 μm sections and subjected to H&E staining assay (Scale bar = 50 μm).

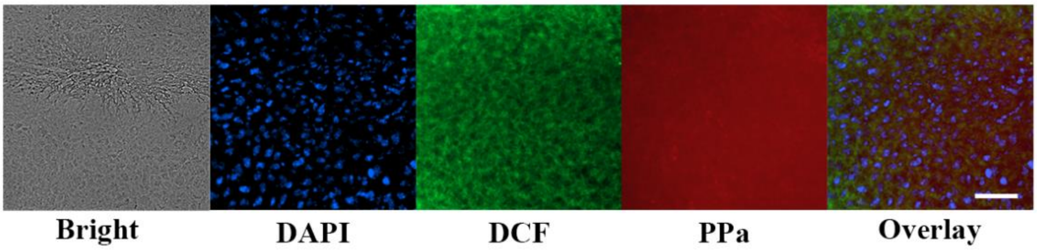

Figure S24. CLSM examination of ROS generation in CT26 xenograft tumor in vivo. The mice were intravenously (i.v.) injected with the HCNSP nanoparticles at a PPa dose of 5.0 mg/kg when the tumor volume reached 200-300 mm^3. Twenty-four hours later, the mice were intratumorally injected with DCFH-DA at a dose of 2.5 mg/kg. Thirty minutes later, the tumor was irradiated with a 671 nm laser for 5 min at a photodensity of 400 mW/cm^2. The tumor was collected, frozen sectioned at 5.0 μm thickness, stained with DAPI and observed by CLSM (scale bar = 50 μm).

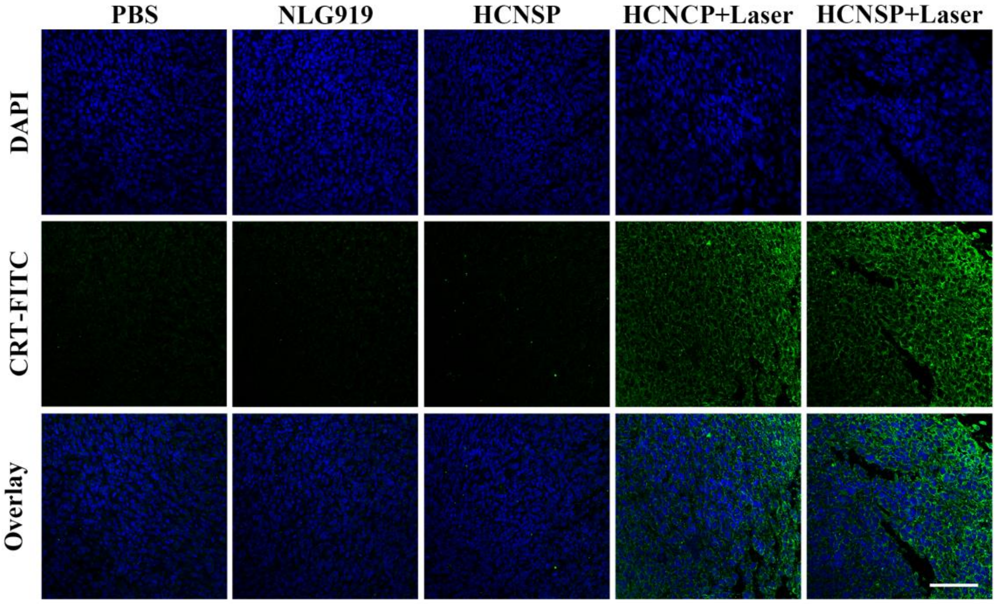

Figure S25. Immunofluorescence examination of CRT expression on the surface of the tumor cells in vivo (scale bar = 100 μm). The mice were treated with NLG919, HCNSP or HCNSP at an identical PPa dose of 5.0 mg/kg or NLG919 dose of 2.5 mg/kg (i.v. injection). The mice of the HCNCP+Laser and HCNSP+Laser group were irradiated with 671 nm laser for 5 min at photodensity of 200 mW/cm^2 at 24h post-injection of prodrug nanovectors. Three days after the treatments, tumors were harvested and fixed in 10% formalin solution, dehydrated, sliced into 5.0 μm sections and stained for anti-CRT fluorescence staining assay.

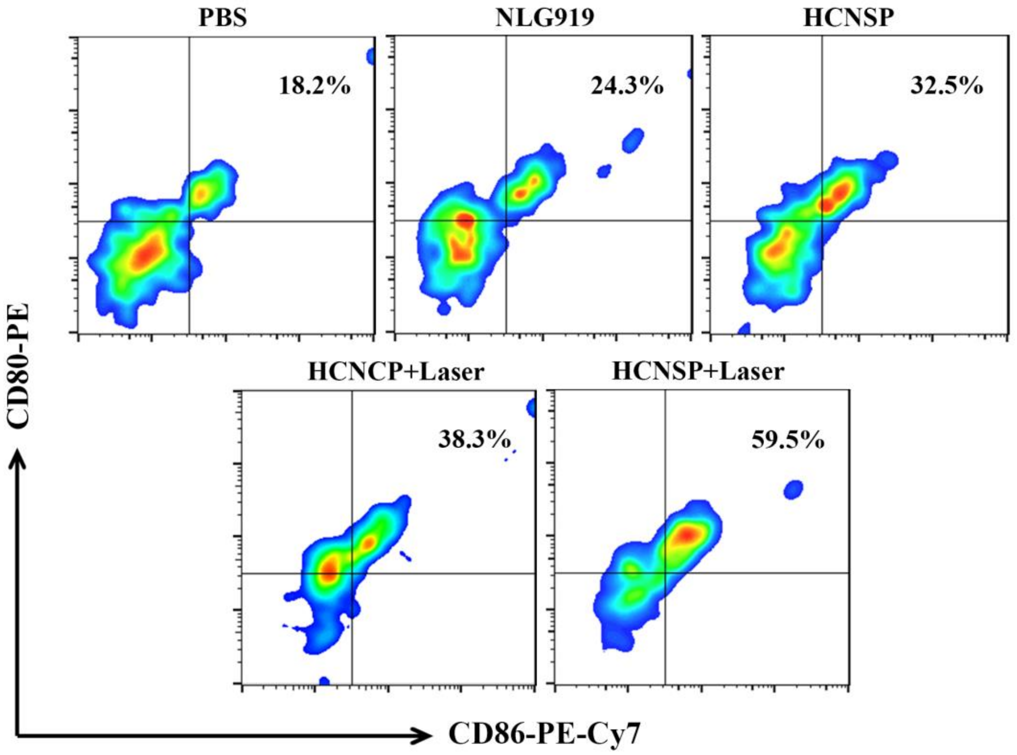

Figure S26. Flow cytometry of DC maturation (gated on CD11c+CD80+CD86+) in the draining lymph nodes of CT26 tumor bearing Balb/c mice receiving different treatments (n = 3). The mice were treated with NLG919, HCNSP or HCNSP at an identical PPa dose of 5.0 mg/kg or NLG919 dose of 2.5 mg/kg (i.v. injection). Twenty-four hours pi, the tumors in HCNCP+Laser and HCNSP+Laser groups were locally irradiated with a 671 nm laser at photodensity of 200 mW/cm^2 for 5 min. DC maturation was determined 3-days following the treatment.

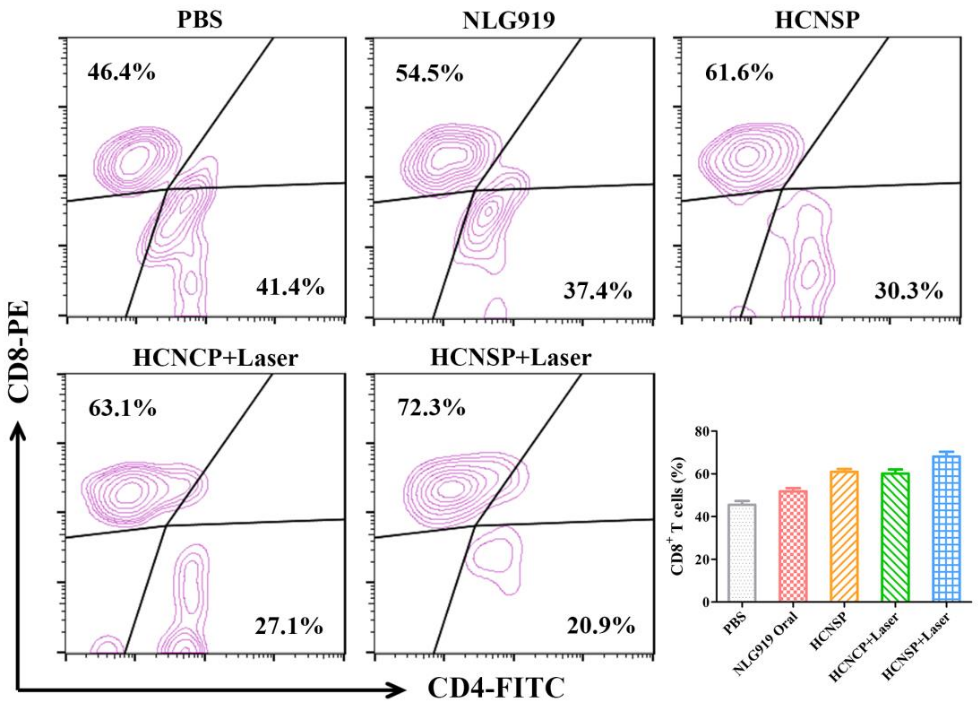

Figure S27. Flow cytometric examination of the intratumoral infiltering $CD8^+$ T cells (gated on $CD3^+CD8^+$) of CT26 tumor bearing Balb/c mice receiving different treatments (n = 3). The mice were treated with NLG919, HCNSP or HCNSP at an identical PPa dose of 5.0 mg/kg or NLG919 dose of 2.5 mg/kg (i.v. injection). The mice in HCNCP+Laser and HCNSP+Laser group were irradiated with 671nm laser for 5 min at photodensity of 200 mW/cm^2 at 24h post-injection. Five days after the treatments, tumors were harvested and cut into small pieces and immersed in the solution of 1 mg/ml collagenase IV and 0.2 mg/ml DNase I for 45 min at 37 ℃ to obtain single cell suspension. Finally, the cells were stained with anti-CD3-PerCP-Cy5.5, anti-CD4-FITC and anti-CD8-PE antibodies for flow cytometric examination.

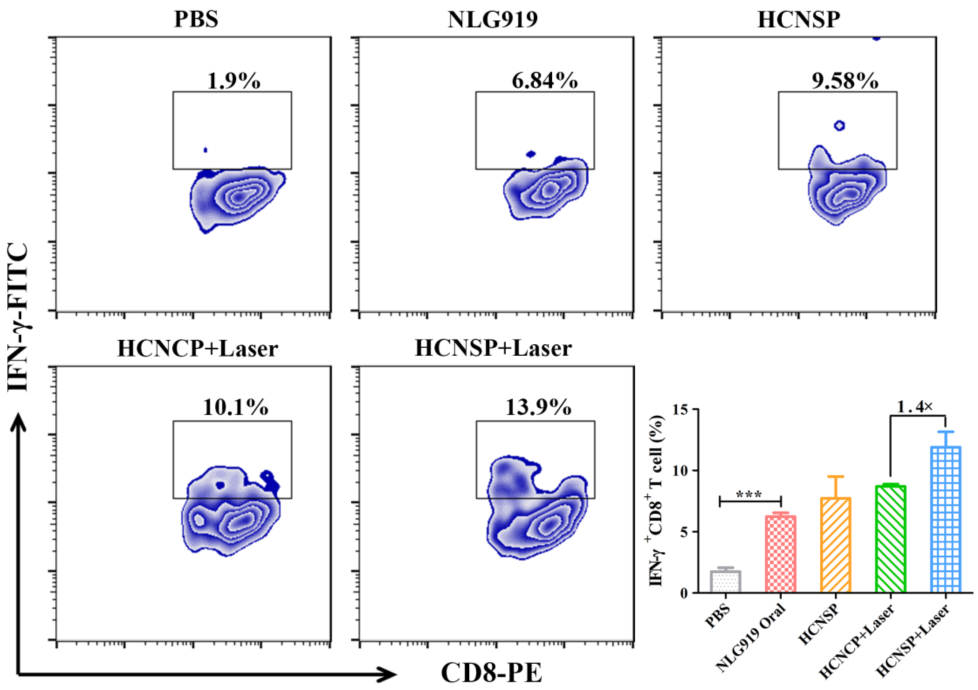

Figure S28. The intratumoral infiltration of IFN-γ$^+$ CTL in the CT26 tumor bearing Balb/c mice receiving different treatments (n = 3). The mice were treated with NLG919, HCNSP or HCNSP at an identical PPa dose of 5.0 mg/kg or NLG919 dose of 2.5 mg/kg (i.v. injection). The mice in HCNCP+Laser and HCNSP+Laser group were irradiated with 671nm laser for 5 min at photodensity of 200 mW/cm^2 at 24h post-injection. Five days after the treatments, tumors were harvested and cut into small pieces and immersed in the solution of 1 mg/ml collagenase IV and 0.2 mg/ml DNase I for 45 min at 37 ℃ to obtain a single cell suspension solution. Finally, the cells were stained with anti-CD3-PerCP-Cy5.5, anti-CD8-PE and anti-IFN-γ-FITC antibodies according to the instructions.

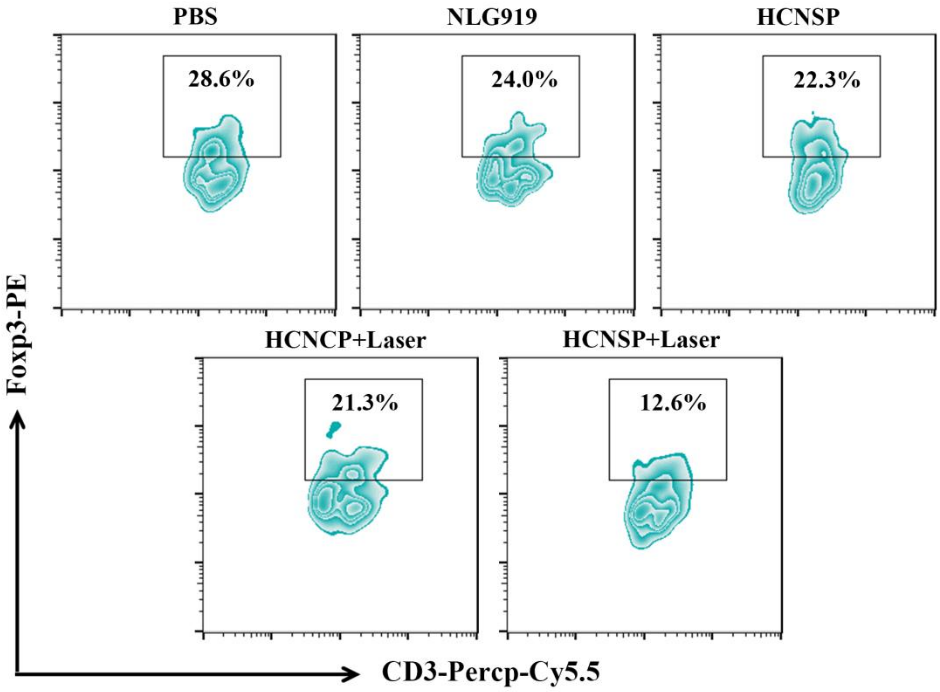

Figure S29. The intratumoral infiltration of Treg in the CT26 tumor bearing Balb/c mice receiving different treatments (n = 3). The mice were treated with NLG919, HCNSP or HCNSP at an identical PPa dose of 5.0 mg/kg or NLG919 dose of 2.5 mg/kg (i.v. injection). The mice in HCNCP+Laser and HCNSP+Laser group were irradiated with 671 nm laser for 5 min at photodensity of 200 mW/cm^2 at 24h post-injection of prodrug nanovectors. Five days after the treatments, tumors were harvested and cut into small pieces and immersed in the solution of 1 mg/ml collagenase IV and 0.2 mg/ml DNase I for 45 min at 37 ℃ to obtain a single cell suspension solution. Finally, the cells were stained with anti-CD3-PerCP-Cy5.5, anti-CD4-FITC and anti-Foxp3-PE antibodies according to the instructions.

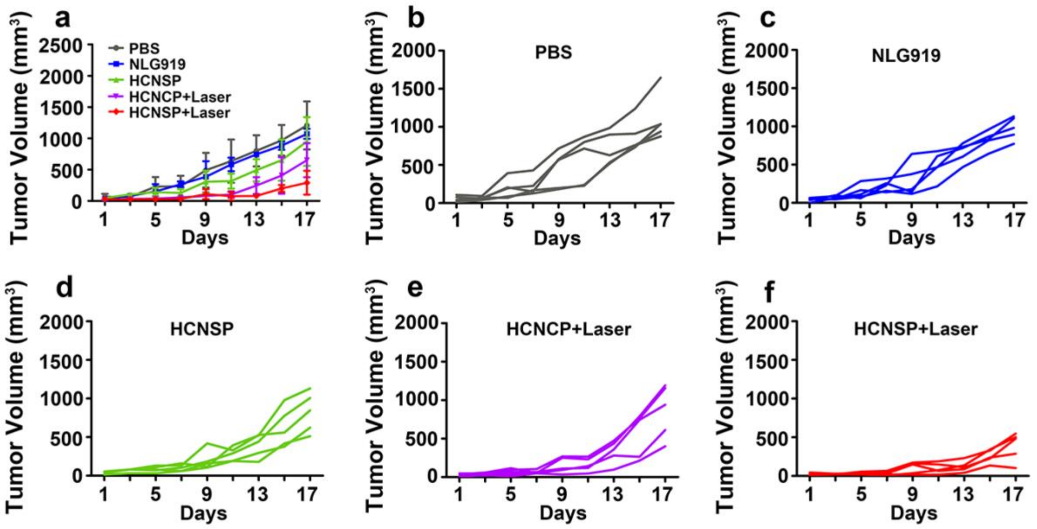

Figure S30. Abscopal tumor growth curves in mice bearing CT26 tumors following the indicated treatments and the specific growth curve of each mouse in each group during the study on abscopal antitumor therapy (n = 5).

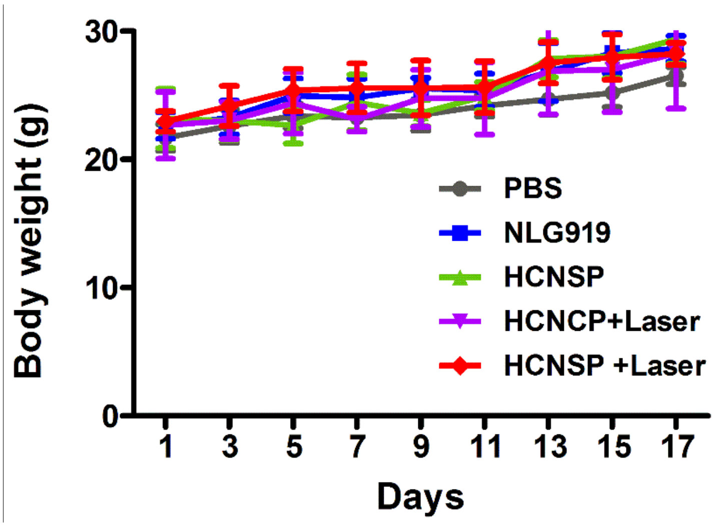

Figure S31. Body weight change of CT26 tumor bearing mice following the indicated treatments during the study on abscopal antitumor therapy (n = 5).

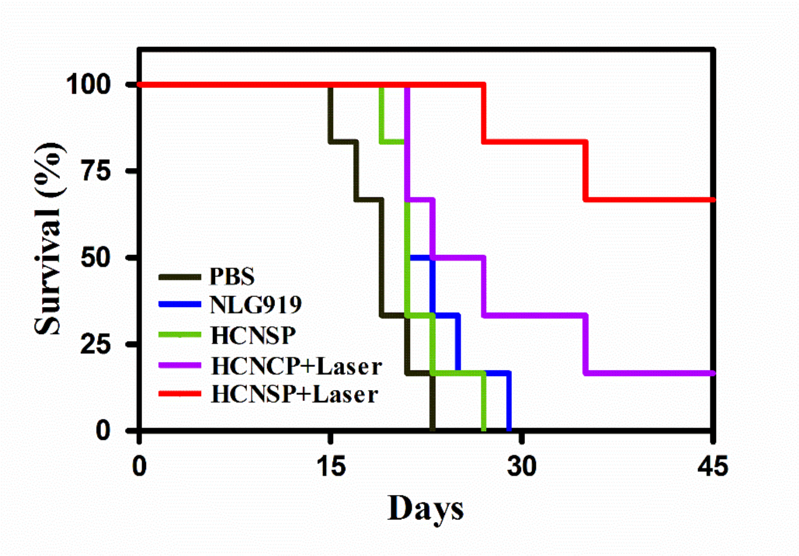

Figure S32. The survival curve of CT26 tumor bearing mice following the indicated treatments during the study on abscopal antitumor therapy (n =5).

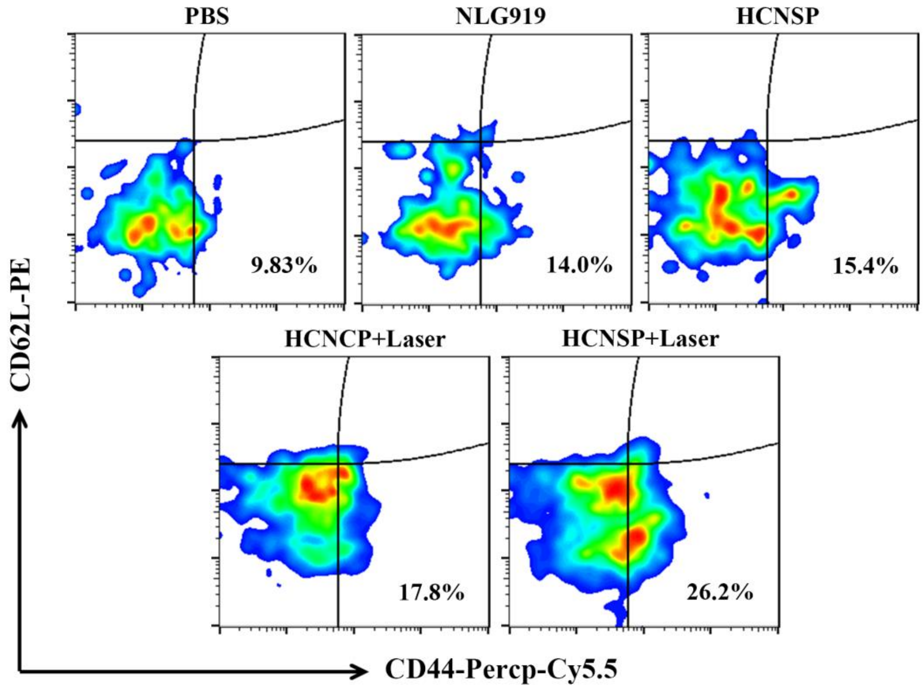

Figure S33. Flow cytometry analysis of memory T lymphocyte in spleen ($CD8^{+}CD44^{+}CD62L^{-}$) of CT26 tumor-bearing Balb/c mice after 30 days treatment (n = 3). The spleen was gently pressed with the piston of syringe, and the single cell suspension was obtained by filtering membrane. Then the single cells were stained with anti-CD8-FITC, anti-CD44-PerCP-Cy5.5 and anti-CD62L-PE antibodies according to the instructions to analyze the T_{EM} ($CD8^{+}CD44^{+}CD62L^{-}$).
